# Construction and Validation of an Immune-Related Prognostic Model Based on TP53 Status in Colorectal Cancer

**DOI:** 10.3390/cancers11111722

**Published:** 2019-11-04

**Authors:** Xiaojuan Zhao, Jianzhong Liu, Shuzhen Liu, Fangfang Yang, Erfei Chen

**Affiliations:** 1 Institute of Preventive Genomic Medicine, School of Life Sciences, Northwest University, Xi’an 710069, China; xjzhao1992@163.com (X.Z.); LSZ15709483140@163.com (S.L.); 2 Key Laboratory of Resource Biology and Biotechnology in Western China, Ministry of Education, School of Life Sciences, Northwest University, Xi’an 710069, China; 3 College of Environmental and Resource Science, Shanxi University, Taiyuan 030000, China; lliujzzhao@163.com

**Keywords:** colorectal cancer, TP53, immunoscore, prognosis, CIBERSORT

## Abstract

Growing evidence has indicated that prognostic biomarkers have a pivotal role in tumor and immunity biological processes. TP53 mutation can cause a range of changes in immune response, progression, and prognosis of colorectal cancer (CRC). Thus, we aim to build an immunoscore prognostic model that may enhance the prognosis of CRC from an immunological perspective. We estimated the proportion of immune cells in the GSE39582 public dataset using the CIBERSORT (Cell type identification by estimating relative subset of known RNA transcripts) algorithm. Prognostic genes that were used to establish the immunoscore model were generated by the LASSO (Least absolute shrinkage and selection operator) Cox regression model. We established and validated the immunoscore model in GEO (Gene Expression Omnibus) and TCGA (The Cancer Genome Atlas) cohorts, respectively; significant differences of overall survival analysis were found between the low and high immunoscore groups or TP53 subgroups. In the multivariable Cox analysis, we observed that the immunoscore was an independent prognostic factor both in the GEO cohort (HR (Hazard ratio) 1.76, 95% CI (confidence intervals): 1.26–2.46) and the TCGA cohort (HR 1.95, 95% CI: 1.20–3.18). Furthermore, we established a nomogram for clinical application, and the results suggest that the nomogram is a better predictive model for prognosis than immunoscore or TNM staging.

## 1. Introduction

TP53, one of the most common tumor suppressor genes, both in colorectal cancer (CRC) and other tumor types, has been well known to play an important role in tumor progression and malignant phenotype of CRC. Although novel driver genes are constantly found in colorectal cancer [1], TP53 alteration is still a main characterization of genetic spectrum in human CRC. Over the past decades, the effects of TP53 as a driver gene in the genomic and biological processes of tumor cells have been widely investigated. Cell cycle arrest, cell apoptosis, and cell migration are the relatively thorough downstream effects of TP53 activity. However, growing evidence suggests that TP53 concurrently contributes to the regulation of tumor immune response [2,3,4,5,6]; a significant activation of inflammatory and innate immune pathways in CRC caused by TP53 mutation have already been found [7]. TP53 can promote immune response by directly activating key regulatory factors in immune signaling pathways [2]. Several TP53 target genes currently have found function in cytokine production and inflammation pathways [8,9,10,11].

In this study, we aim to construct a prognostic model in CRC, which is a gene combination. The genes in this model might be regulated by TP53 and closely related to immune infiltration. In order to obtain immunological genes, CIBERSORT (Cell type identification by estimating relative subset of known RNA transcripts) [12], a freely available tool, was used to quantify the relative proportions of immune cell types, including B cells, T cells, NK cells, macrophages, and myeloid subsets. To accurately estimate immune cell subtype, LM22 was used as an input file of reference, which is a gene expression matrix. We also performed least absolute shrinkage and selection operator (LASSO) with L1-penalty to select a gene set that has the most prognostic value to establish an immunoscore model.

## 2. Results

### 2.1. Relationship of Immune Status and TP53 Mutations in CRC Patients

**Figure 1:**
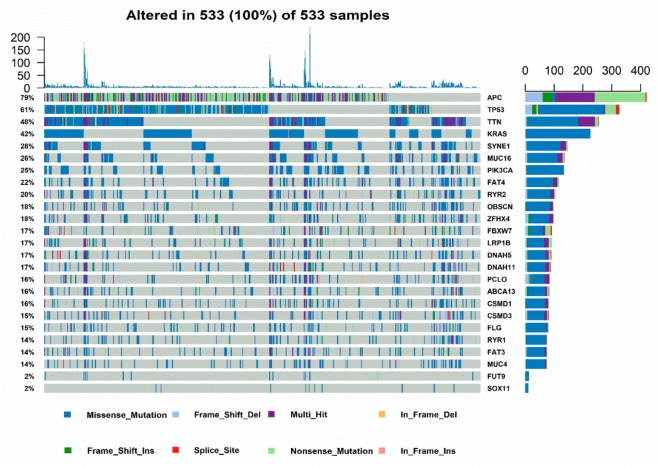

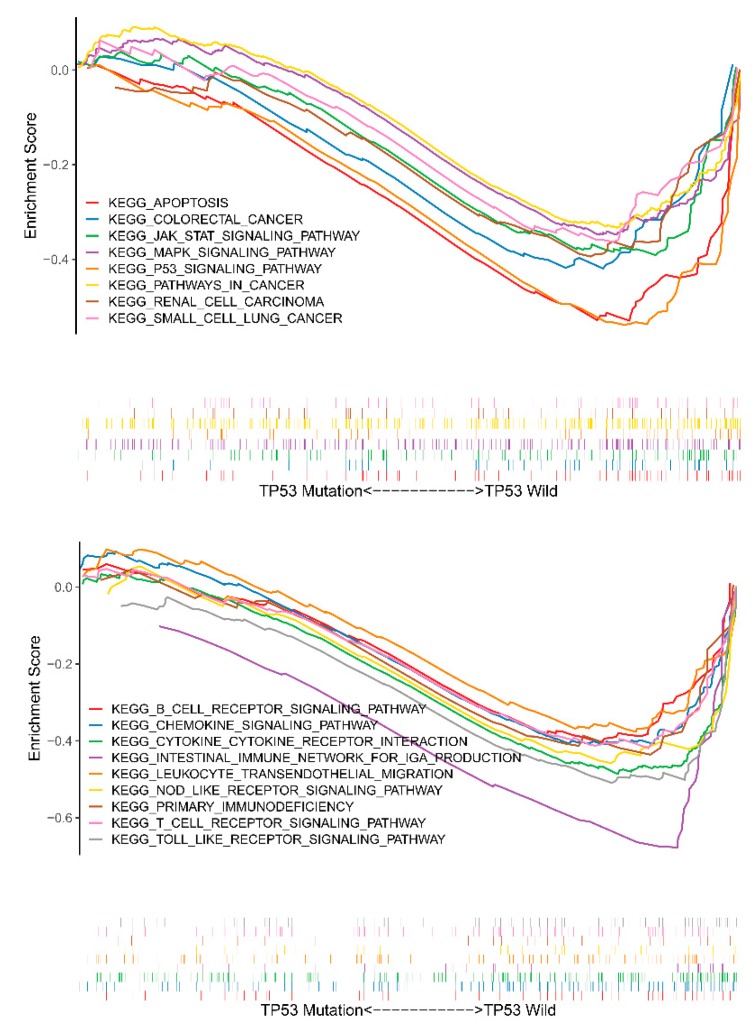
Genomic landscape of CRC and gene set enrichment analysis of CRC samples based on TP53 status in the TCGA (The Cancer Genome Atlas) cohort. (**a**) Frequency and type of mutations in the top 20 genes in CRC. Genes are sorted according to frequency of mutations. (**b**,**c**) The immunity and cancer pathways that are significantly enriched in TP53^WT^ CRC patients compared with that in TP53^MUT^ CRC patients.

TP53 mutation is one of the most common type of mutations in CRC based on TCGA cohort, ranked only second to APC (Figure 1a). The Kaplan-Meier analysis showed that there is no significant difference between TP53 mutation status and overall survival in patients with CRC in GEO and TCGA cohorts, which is consistent with previous research [13] (Supplemental Figure S1a,b). Although the results of OS were not significant, the role of TP53 in CRC survival still needs further investigation.

We subsequently divided CRC samples in the GEO cohort into TP53^WT^ (161 samples) and TP53^MUT^ (190 samples) groups, and performed Gene Set Enrichment Analysis (GSEA) analysis. The results showed that TP53^WT^ CRCs were significantly enriched in 61 KEGG pathways (*p* < 0.05) (Supplemental Table S1), including pathways that were involved in immune signaling: T-cell-receptor-signaling-pathway, B-cell-receptor-signaling-pathway, intestinal-immune-network-for-IGA-production, and tumor proliferation, apoptosis and migration related signaling pathways, such as JAK-STAT-signaling-pathway, apoptosis, MAPK-signaling-pathway, and P53-signaling-pathway (Figure 1b,c). On the contrary, TP53^MUT^ CRCs were not enriched in any immune and cancer-related signaling pathways (Supplemental Table S2). These findings indicate that TP53 mutation not only affects the proliferation, apoptosis, and migration of CRC, but also plays a crucial role in immune response. Additionally, DEGs linked to TP53 status were acquired by *limma* package between TP53^WT^ and TP53^MUT^ samples, as shown in Supplemental Table S3.

### 2.2. Immune Landscape and Immune Cell Subset in CRC

To assess immune landscape and identify immune-related genes, the GSE39582 cohort was selected in this study. Among our candidate datasets, GSE39582 had the largest number of samples with detailed sample information, including TP53 status. More importantly, the CIBERSORT algorithm was more suitable to data from the Affymetrix platform. The immune landscape of immune cell subset proportions is detailed in Supplemental Figure S2.

**Figure 2:**
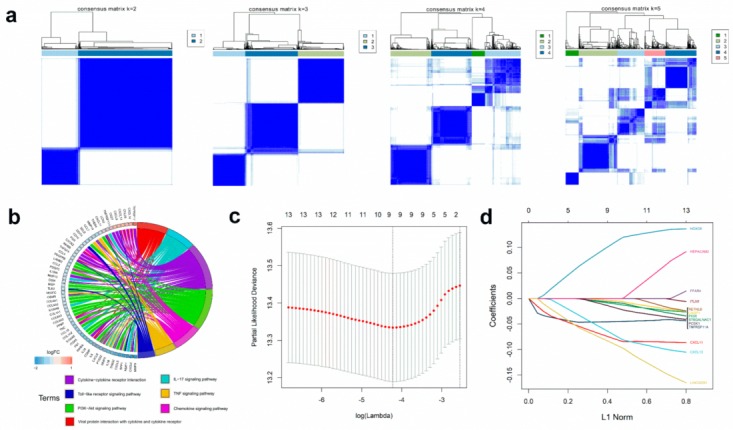

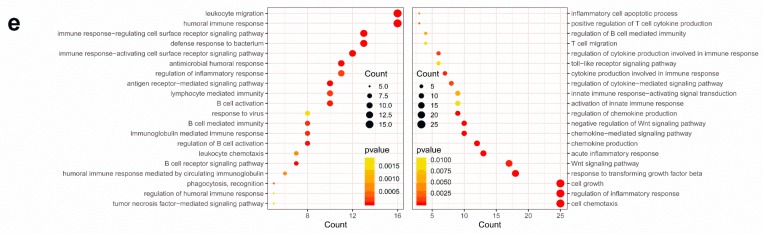
Immune-related DEGs and construction of the immunoscore model. (**a**) Consensus matrixes of CRC samples in GEO cohort for each k (*k* = 2–5), using 1,000 iterations of hierarchical clustering. (**b**) Kyoto encyclopedia of genes and genomes (KEGG) analysis of immune-related DEGs (Differentially expressed genes). Circular plot of KEGG pathways was enriched, which was related with immune response and tumor proliferation, apoptosis, and migration. (**c**,**d**) LASSO coefficient profiles of 13 genes were related with prognostics. The optimal values of the penalty parameter λ were determined by ten-fold cross-validation. (**e**) Gene Ontology (GO) analysis of the immune-related DEGs. The immunity and cancer biological processes significant were enriched from immune-related DEGs. The processes in the left figure were enriched from up-regulated DEGs. The processes in the right figure were enriched from the up-regulated DEGs.

Two distinct patterns of CRC samples were revealed by cluster analysis, as shown in Figure 2a. We identified immune-related DEGs (Supplemental Table S4); GO enrichment analysis revealed that DEGs were involved in B cell, T cell regulation, and cancer induction (Figure 2e). Figure 2b indicated that DEGs participate in tumor necrosis factor (TNF), chemokine, interleukin, PI3K-Akt e.g., signaling pathways. Details of the enrichment analysis were shown in Supplemental Figure S3 and Supplemental Table S5.

### 2.3. Calculation of the Immunoscore and Evaluation of Its Prognostic Ability

We identified the overlapping genes between TP53 status-related DEGs (199 genes, shown in Supplemental Table S3) and immune phenotype-related DEGs (438 genes, shown in Supplemental Table S4). Univariate Cox regression analysis revealed that 13 of the 37 overlapping genes were significantly related to OS status, and 9 genes with maximum prognostic value were found using LASSO Cox regression analysis (Figure 2c,d). Finally, we selected these genes to build an immunoscore model to evaluate the prognostic ability of CRC patients. The formula for the immunoscore model was described in Materials and Methods. The correlation between immunoscore and constructed genes is shown in Supplemental Figure S4.

**Figure 3:**
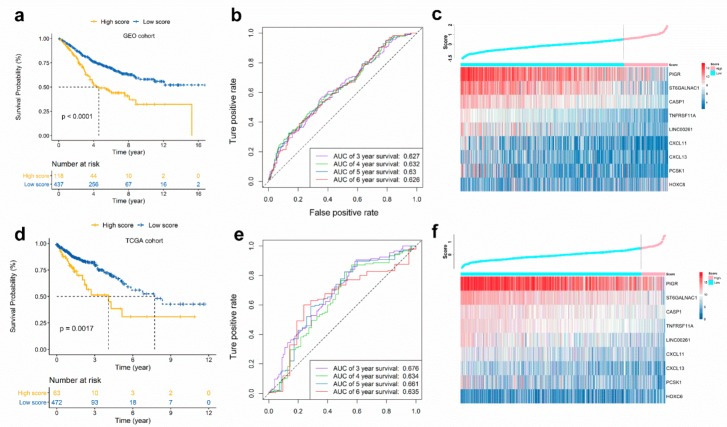
Validation of immunoscore model. Patients were stratified by low or high immunoscore (low and high score). Kaplan-Meier curves, heatmap and time-dependent ROC curve in GEO cohort (**a**–**c**) and TCGA cohort (**d**–**f**). (**a**,**d**) Kaplan-Meier curves show that OS in the low score group was significantly higher than in the high score group. (**b**,**e**) Time-dependent ROC curve analysis of the immunoscore. (**c**,**f**) Relationship between the immunoscore and the heatmap for the expression of the selected prognostic genes.

Next, we categorized CRC patients into high or low score groups, according to the optimal cut-off value of immunoscore obtained from *survminer* R package. The results showed that high score patients had a worse OS than those of low score patients (hazard ratio (HR) 2.09, 95% confidence interval (CI): 1.53–2.85, *p* < 0.001) (Figure 3a). We also obtained similar results in disease-free survival (DFS) analysis (Supplemental Figure S5). Figure 3b showed the prognostic accuracy of immunoscore, which was investigated as a continuous variable. The area under the ROC curves (AUC) of the prognostic model for OS was 0.627 at 3 years, 0.632 at 4 years, 0.630 at 5 years, and 0.626 at 6 years. Figure 3c showed the immunoscore distribution and selected gene expression data.

### 2.4. Validation of the Immunoscore in TCGA CRC Cohort

To determine if the immunoscore model is solid in different populations, we performed an identical formula and identified a cut-off value in the TCGA CRC cohort. The patients were then divided into high- or low-risk groups. Consistent with the findings in the GEO CRC cohort, patients categorized into the high score group had a lower OS rate than the low score group (HR 2.13, 95% CI: 1.31–3.45, *p* < 0.005) (Figure 3d). The results demonstrated that the predictive potential of the immunoscore model is applicable in different populations and different platforms. The prognostic accuracy of the immunoscore in the TCGA cohort was also evaluated; the AUC achieved 0.676, 0.634, 0.661, and 0.635 at 3, 4, 5, and 6 years, respectively (Figure 3e). Figure 3f showed the risk score distribution and selected genes expression data.

Furthermore, we observed that the immunoscore, as a continuous variable in univariate Cox regression analysis and multivariable Cox analysis, was an independent prognostic factor, both in the GEO cohort (HR 1.76, 95% CI: 1.26–2.46) and the TCGA cohort (HR 1.95, 95% CI: 1.20–3.18) (Supplemental Tables S6 and S7).

### 2.5. Survival Analysis for the Immunoscore According to TP53 Status

**Figure 4:**
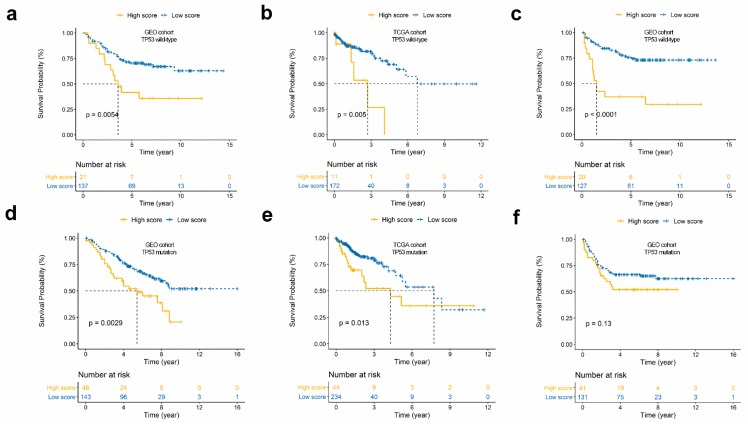

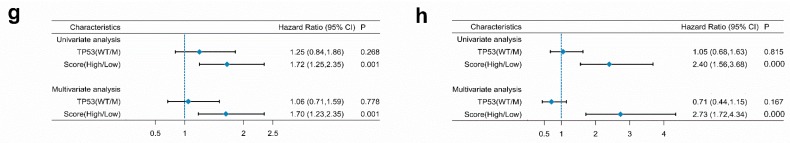
Prognosis analysis of the TP53 subgroup. (**a**,**b**) Overall survival curves of TP53^WT^ subgroups in the GEO (**a**) and TCGA cohort (**b**). (**d**,**e**) Kaplan-Meier curves of TP53^M^ subgroups in the GEO (**d**) and TCGA cohort (**e**). (**c**,**f**) Disease-free survival curves of TP53^WT^ (**c**) and TP53^M^ (**f**) subgroups in the GEO cohort. (**g**,**h**) Univariate and multivariate regression analysis of the relation between the immunoscore and TP53 status regarding prognostic value in the GEO cohort (**g**) and TCGA cohort (**h**).

To investigate if the prognostic potential of the immunoscore is independent of TP53 status, we performed a stratification analysis. Patients in the GEO cohort and the TCGA cohort were assigned to two groups on the basis of TP53 status; the stratification analysis results suggest that there is a significant relationship between immunoscore and OS in the TP53^WT^ and TP53^MUT^ groups—the high score group had a worse OS rate than the low score group, not merely in the GEO cohort (Figure 4a,d), but in the TCGA cohort as well (Figure 4b,e). Moreover, DFS analysis results for the GEO cohort showed a significant difference in the TP53^WT^ group (Figure 4c), but not in the TP53^MUT^ group (Figure 4f).

Additionally, by applying univariate and multivariate Cox regression analyses, we demonstrated that the predictive value of the immunoscore for patients with CRC is independent of TP53 status (Figure 4g,h).

### 2.6. Immune Landscape Between Low and High Score CRC Patients

**Figure 5:**
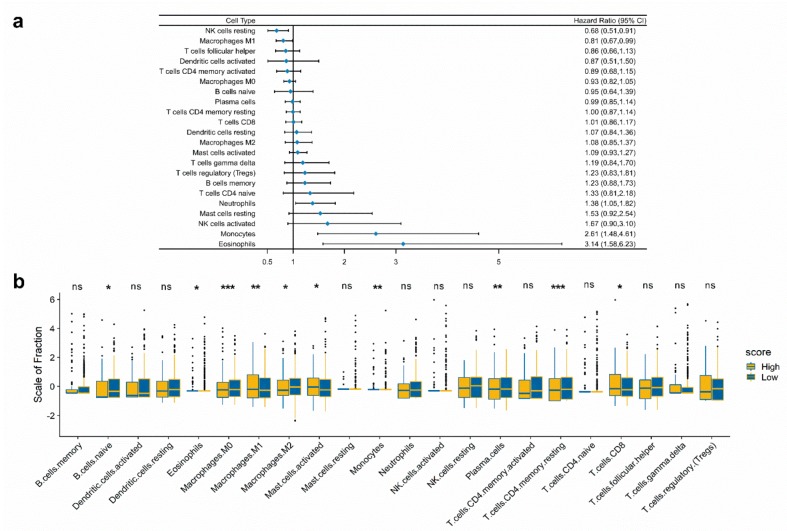
The landscape of immune infiltration in high and low immunoscore groups of CRC patients. (**a**) Forest plots showing associations between different immune cell subsets and overall survival in the GEO cohort. (**b**) The fraction of immune cell types in low and high immunoscore groups. The statistical differences of two groups were compared through the unpaired *t* test. The *p* values are labeled above each boxplot with asterisks. (* *p* < 0.05, ** *p* < 0.01, *** *p* < 0.001, **** *p* < 0.0001).

Using the CIBERSORT method, we estimated the fraction of immune cell types in CRC patients; the results are summarized in Supplemental Figure S2. The proportion of immune cells varies between the CRC samples. We speculated that variations in the proportions of tumor-infiltrating immune cells might be an intrinsic feature representing individual differences. We normalized the proportion levels of immune cells with mean value = 0 and standard deviation (SD) = 1. A forest plot based on univariable Cox analysis showed the relationship between immune cell types and overall survival (Figure 5a). We compared the proportions of immune cells between low and high score CRC patients—significant differences were found in B cells naive, Plasma cells, T cells CD8, T cells CD4 memory resting, Monocytes, Macrophages M0, Macrophages M1, Macrophages M2, Mast cells activated, and Eosinophils (Figure 5b, Supplemental Table S8). These differences between groups indicated that the variations in proportions of tumor-immune cells might be correlated with the CRC samples’ overall survival. Therefore, immune infiltration heterogeneity in CRC may be used as a prognosis indicator, which has remarkable and practical clinical implications.

### 2.7. Construction and Validation of the Nomogram 

**Figure 6:**
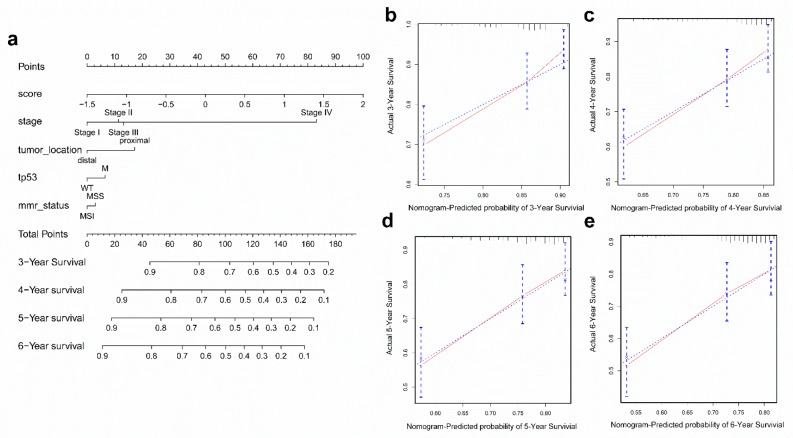
Construction and validation of a nomogram. (**a**) Nomogram to predict the probability of OS in 3, 4, 5, and 6 years for CRC in the GEO cohort. (**b**–**e**) Calibration plot of the nomogram to predict the probability of OS at 3, 4, 5, and 6 years.

To further improve the accuracy of the prognostic, we constructed a nomogram that integrated immunoscore and clinical information, including TP53 status, tumor stage, tumor location, and microsatellite status to quantitatively predict the prognosis of CRC patients in the GEO cohort. In this nomogram, the score for each variable can be found on the point scale, so it is easy to ascertain the estimate probability of survival at 3, 4, 5, and 6 years by calculating the total score (Figure 6a). Compared to the other clinical factors, immunoscore had the most score points.

To validate the nomogram’s performance, we conducted calibration curves and observed that the predictive curves were close to the ideal curve (Figure 6b–e), indicating good functioning. Moreover, the predictive accuracy of this nomogram (C-index: 0.667) was higher than immunoscore (C-index: 0.612), TP53 status (C-index: 0.516), tumor stage (C-index: 0.593), tumor location (C-index: 0.540), and microsatellite status (C-index: 0.508). These results demonstrated that the nomogram is a better predictive model for survival than a single prognostic factor.

## 3. Discussion 

In colorectal cancer, TP53 mutation can cause a significant activation of inflammatory and innate immune pathways [7]. In HCT116 cells, TP53 influences the expression and function of essential immunoreceptors involved in host defence, such as the Toll-like receptor [14]. These findings indicate that TP53 not only affects the proliferation, apoptosis, and migration of CRC, but also plays a crucial role in immune response. Furthermore, immunoreceptors are also critical for CRC proliferation, invasion, and migration [15,16], and play roles as useful prognostic markers [17]. These comprehensive studies highlight the pivotal function of TP53 and immunoreceptors in the cancer and immunity process. Within this context, we aimed to further explore the role of immune-related biomarkers related to TP53 status. Moreover, it is essential to generate a valuable prognostic model constructed with immune-related biomarkers, which could improve the efficacy of immunotherapy in stratified CRC patients. 

In this retrospective analysis, we established and verified an immune score prediction model based on TP53 mutation status and immunity to improve the accuracy of CRC prognosis, namely, immunoscore, which is a set of differentially expressed genes related to prognosis of CRC. The results of overall survival analysis and disease-free survival analysis show a significant statistical difference between CRC patients with high and low immunoscore. In addition, the immunoscore was still an independent prognostic factor after multivariable adjustment for clinical characteristics. These results demonstrated that immunoscore in this research had the similar potential of prognostic accuracy as traditional prognostic factors.

In current years, the crucial role of host immune response against cancer has been investigated, and the prognostic assessment of the in situ immune cell infiltrate in tumor progression has been demonstrated [18]. Several types of immunoscore models have been developed and verified, and the scores were based on numeration lymphocytes in tumor regions [19], ratio of immune cells [20], or expression of prognostic genes [21]. However, current studies are only focused on the immune response status. In this study, we took into account the role of TP53 mutation status in prognostic biomarkers in CRC. In a GSEA analysis for CRC samples with and without TP53 mutation, we found that immune signaling pathways and cancer pathways were obviously enriched, such as the Toll-like receptor signaling pathway. We then selected the downstream effect genes of TP53 and immune-related genes. Unlike previous studies [21,22], the immune-related genes selected in our model were determined by immune cell proportion generated by the algorithm CIBERSORT, which is an analytical method suitable for gene expression profile data from GEO public databases. By applying cluster analysis to immune cell proportion data of CRC, the samples were steadily clustered to groups and immune-related genes generated from two subgroups by *limma* R package. This method had already been implemented and verified for gastric cancer [23]. The immune-related genes were significantly enriched in immunity biological processes in GO analysis, and enriched in immune signaling pathways and cancer pathways in KEGG analysis. To improve the predictive accuracy of our immunoscore model, we adopted the LASSO Cox regression model because it is suitable for finding genes with the greatest prognostic value [24].

Most immune-related prognostic genes that constituted our immunoscore model were cytokine, cytokine receptor, and transcriptional regulation. Chemokines can mediate inflammation and they are well-known for their role in mediating immune cell trafficking. Previous research had reported that CXCL13 is associated with CRC infiltration by distinct T cell subsets [25], and CXCL11 is a member of the confirmed prognostic model of gastric adenocarcinoma [26]. HOXC6, a member of the homeobox family that encodes highly conserved transcription factors, not only plays a crucial role in CRC [27], but also functions as an independent prognostic marker for hepatocellular carcinoma [28] and prostate cancer [29]. LINC00261 [30,31,32], TNFRSF11A [33], CASP1 [34], ST6GALNAC1 [35], and PIGR [36] also play prognostic roles in cancer. Only PCSK1’s function in cancer and immunity has not been researched. In this study, for the first time, we discovered prognostic immune-related genes that related TP53 status and gathered them to establish an immune prognostic model. Immune-related genes in our established immunoscore model can be regarded as individual biomarkers, and their immune characteristics and prognostic significances may help ensure better performance in clinical combination therapy.

Furthermore, we established a nomogram based on multivariate Cox regression coefficients of immunoscore, TP53 status, tumor stage, tumor location, and microsatellite status. A satisfactory agreement between the observed values was observed in calibration curves. Additionally, the C-index of the nomogram was significantly higher than the C-index of TNM staging, suggesting that the accuracy of prognosis is higher when immune status is taken into account, and immunoscore can be a more accurate prognostic factor than TNM staging. These findings are consistent with previous research that showed that the immunoscore was similar with or higher than TNM staging, which can provide an accurate prognosis of CRC recurrence [18]. The prognostic accuracy is higher with a comprehensive consideration of immunoscore and TNM staging together rather than by themselves. 

Although our research provides new insights into immune-related therapies for CRC, there are limitations to our study. First, the datasets in our prognostic model are based on a public database. Therefore, the information provided is limited, and more detailed clinical information could not be acquired to improve prognostic accuracy. Thus, further prospective studies are needed on the subject. 

## 4. Materials and Methods

### 4.1. Microarray Datasets

In order to obtain a gene expression dataset of CRC, 566 CEL files of GSE39582 based on platform GPL570 were downloaded from the Gene Expression Omnibus (GEO) database (https://www.ncbi.nlm.nih.gov/geo/). The Robust Multiarray Averaging (RMA) method in the *Affy* R package was used to process the CEL files. The corresponding clinical data were obtained directly from the relevant gene expression profiles. 

### 4.2. RNA-Sequencing Datasets

The somatic mutation status data (identified by VarScan2), gene expression data, and corresponding clinical information of CRC were downloaded from The Cancer Genome Atlas (TCGA) website (https://portal.gdc.cancer.gov/repository). 536 samples with RNA-sequencing data and TP53 mutation status were subjected to further study. We used the *edger* R package to normalize the RNA-sequencing data; log2 transformations were performed for all expression data. 

### 4.3. Estimation of Immune Cell Type Fractions

The CIBERSORT algorithm has been already verified for gene expression profiles measured using microarrays. In this study, CIBERSORT and LM22 were utilized to quantify the proportion of immune cells in the CRC samples from microarray data. LM22 is a gene signature matrix containing 547 genes that discriminate hematopoietic cell phenotypes. Normalized gene expression data were analyzed using the CIBERSORT algorithm, running with 1,000 permutations. The output included a *p*-value for the deconvolution of each sample using Monte Carlo sampling. The CIBERSORT *p*-value reflects the statistical significant of the result; a threshold <0.05 is recommended. Finally, samples with CIBERSORT *p* < 0.05 were included to identify immune-related DEGs. 

### 4.4. Differentially Expressed Genes (DEGs) Associated with TP53 Status and Immune Phenotype

To cluster 451 CRC samples with CIBERSORT *p* < 0.05 into different groups, we used the *ConsensusClusterPlus* R package to select the optimal cluster number. Moreover, 351 CRC samples with TP53 information were divided into groups with and without TP53 mutation. We used the *limma* R package to determine immune DEGs, which are also related to TP53 status.

### 4.5. Functional and Pathway Enrichment Analysis

In order to investigate biological pathways correlated with immunity and cancer between TP53^WT^ (*n* = 161) and TP53^M^ (*n* = 190) groups, we performed GSEA (Version: 4.0) analysis. We selected C2.CP: KEGG.V7.0.symbols.gmt file as the reference gene set file. The threshold was set at *p* < 0.05.

We used the *clusterProfiler* R package to perform gene annotation enrichment analysis on immune-related genes. Gene Ontology (GO) analysis identified biological processes in up- and down-regulated gene sets. Pathways enriched in immune-related gene set were identified by Kyoto Encyclopedia of Genes and Genomes (KEGG) analysis. GO and KEGG analysis were using the cutoff of *p* < 0.05, false discovery rate (FDR) of <0.05. 

### 4.6. Construction and Validation of an Immunoscore Prognostic Model 

By using the univariable Cox proportional hazards regression model, we calculated the hazard proportions for DEGs of the GEO cohort. DEGs with significance at *p* < 0.05 were analyzed, and we used LASSO to select the most useful prognostic genes among DEGs. A formula for the immunoscore model was established to predict patient survival:

immunoscore = Σ Cox coefficient of gene Xi × scale expression value of gene Xi

### 4.7. Construction and Validation of a Nomogram Model

We used multivariate Cox regression coefficients of clinical characteristics to establish a nomogram that visualizes the prognostic value of different risk scores in a single figure. This analysis was performed by the *rms* R package by plotting calibration curves to assess the predicted probabilities, compared with the best predictive line. In addition, Concordance index (C-index) was used to determine the predictive accuracy of the nomogram.

### 4.8. Statistical Analysis

The unpaired *t* test was used to estimate the statistical significance for normally distributed variables of the two groups. The survival curve for the subgroups was generated by the Kaplan-Meier method and the statistical significance of difference was determined by the Log-rank test. The *survminer* package was used to evaluate the optimal cut-off value based on the association between overall survival and immunoscore in each dataset. The univariable Cox proportional hazards regression model was used to calculate a hazard ratio for univariable analysis. In order to select the most useful prognostic genes, we applied the LASSO Cox regression algorithm to the prognosis-associated genes in the GEO cohort. A multivariate Cox regression analysis was done to determine independent prognostic factors; only patients with integrated clinical information were included. The receiver operating characteristic (ROC) curve was used to depict the sensitivity and specificity of survival prediction based on the immunoscore, and the *timeROC* R package was used to quantify the area under the curve (AUC). All statistical analyses were conducted using R software. All statistical tests were two-tailed, with a value of *p* < 0.05 considered statistically significant.

## 5. Conclusions

It was for the first time that an immunoscore prognostic model based on immune-related genes was generated and validated by considering the influence of TP53 mutation status in CRC. It provides a novel immunological perspective to improve clinical therapy performance in CRC.

## Supplementary Materials

The following are available online at https://www.mdpi.com/2072-6694/11/11/1722/s1, Figure S1: Prognostic analysis of TP53 status in CRC, Figure S2: Relative proportion of immune infiltration in GEO CRC patients, Figure S3: Bubble plot of GO analysis results, Figure S4: Correlation of the immunoscore with the expression of genes that constructed immunoscore, Figure S5: disease-free survival (DFS) analysis of immunoscore in GEO cohort, Table S1: GSEA enrichment in TP53^WT^ CRCs, Table S2: GSEA enrichment in TP53^M^ CRCs, Table S3: Differentially expressed genes between TP53^M^ and TP53^WT^ in CRCs, Table S4: Differentially expressed genes between subgroups based on proportion of immune cells in CRC, Table S5: KEGG analysis results of immune-related DEGs in CRC, Table S6: Results of univariate Cox regression analysis, Table S7: Results of multivariable Cox regression analysis, Table S8: The difference of proportion of immune cell types in low and high score CRC groups.
