# Supplementary material for: Construction and Validation of an Immune-Related Prognostic Model Based on TP53 Status in Colorectal Cancer
Source: Cancers (Basel). 2019 Nov 4;11(11):1722. doi: 10.3390/cancers11111722 (PMC6895875; doi:10.3390/cancers11111722)
Supplement: Supplementary file 1 [file cancers-11-01722-s001.pdf]

Supplementary Materials

Construction and Validation of an Immune-Related Prognostic Model Based on TP53 Status in Colorectal Cancer

Xiaojuan Zhao [1,2], Jianzhong Liu [3], Shuzhen Liu [1,2], Fangfang Yang [1,2,*] and Erfei Chen [1,2,*]

Supplementary Figures

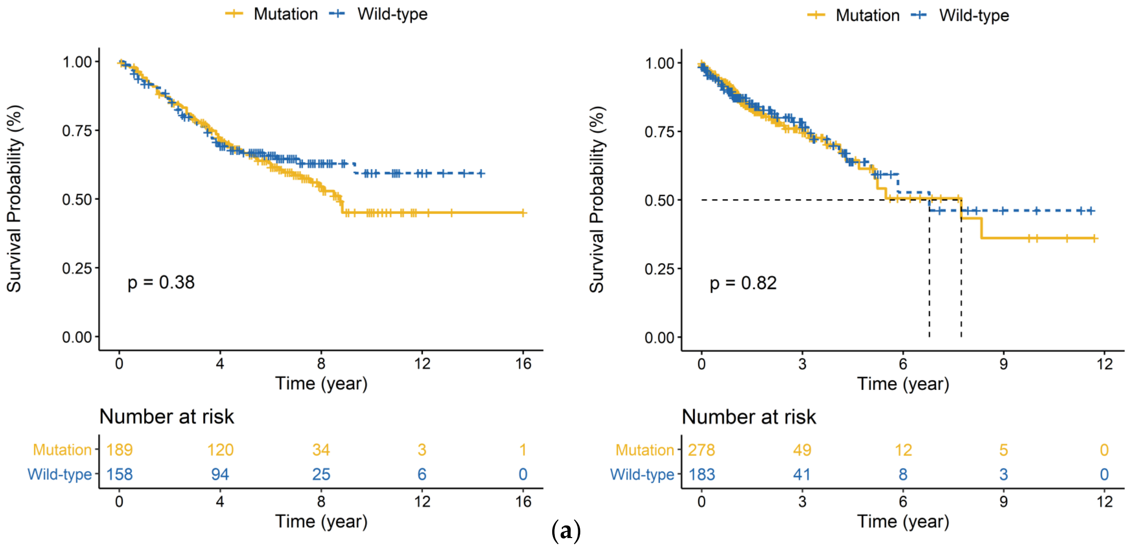

(**a**) (**b**)

Figure S1. Prognostic analysis of TP53 status in CRC. According to TP53 with and without mutation, we stratify CRC patients into groups. Kaplan-Meier curves showed that there were no significant differences in groups, neither in GEO dataset (**a**) nor in TCGA dataset (**b**).

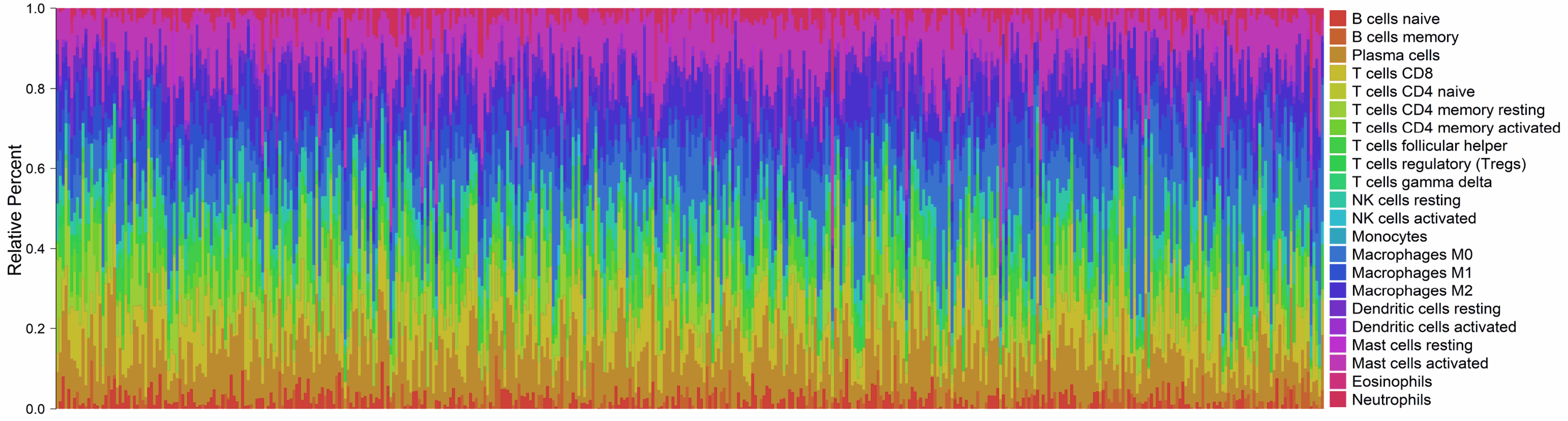

Figure S2. Relative proportion of immune infiltration in GEO CRC patients. CRC samples with CIBERSORT $p < 0.05$ were selected to further investigation, the relative proportion of 22 immune cell types as shown in figure. The total of proportions of immune cell types in every CRC sample is 1.0. Obviously, the proportions of immune cells in CRC samples varies.

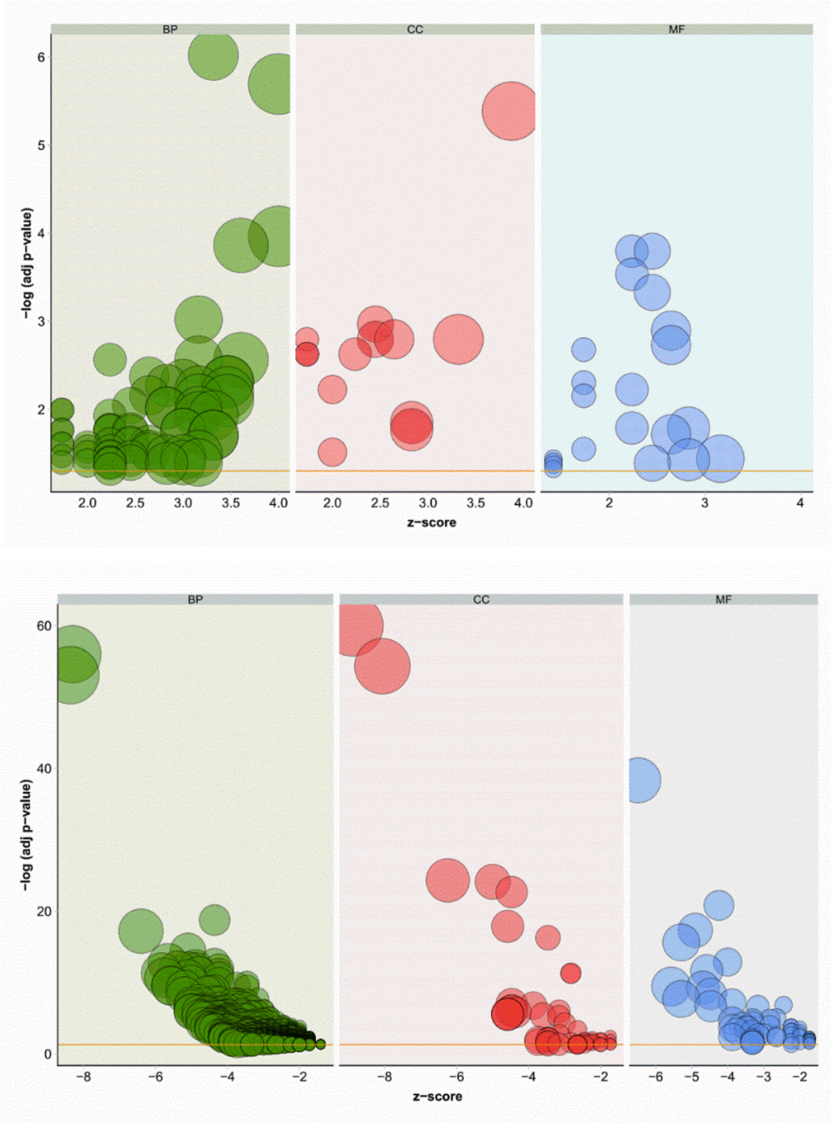

Figure S3. Bubble plot of GO analysis results. **a** is the bubble plot of GO analysis results for up regulation genes, **b** is the bubble plot of GO analysis results for down regulation genes. X-axis: zscore = (up–down)/ √ count, *up* and *down* are the number of assigned genes up-regulated (logFC > 0) in the data or down- regulated (logFC < 0), respectively. Count is the number of genes assigned to a term. Y-axis: $-\log_{10}(p\text{-value})$.

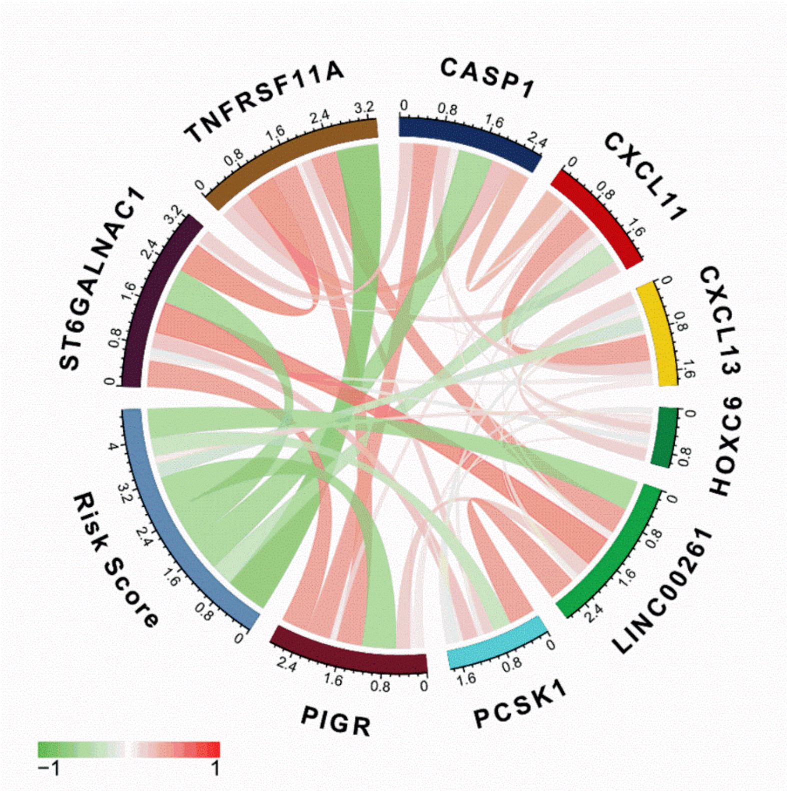

Figure S4. Correlation of the immunoscore with the expression of genes that constructed immunoscore. Red connecting line represents a positive correlation, green connecting line represents a negative correlation. The darker the color, represents the greater the correlation.

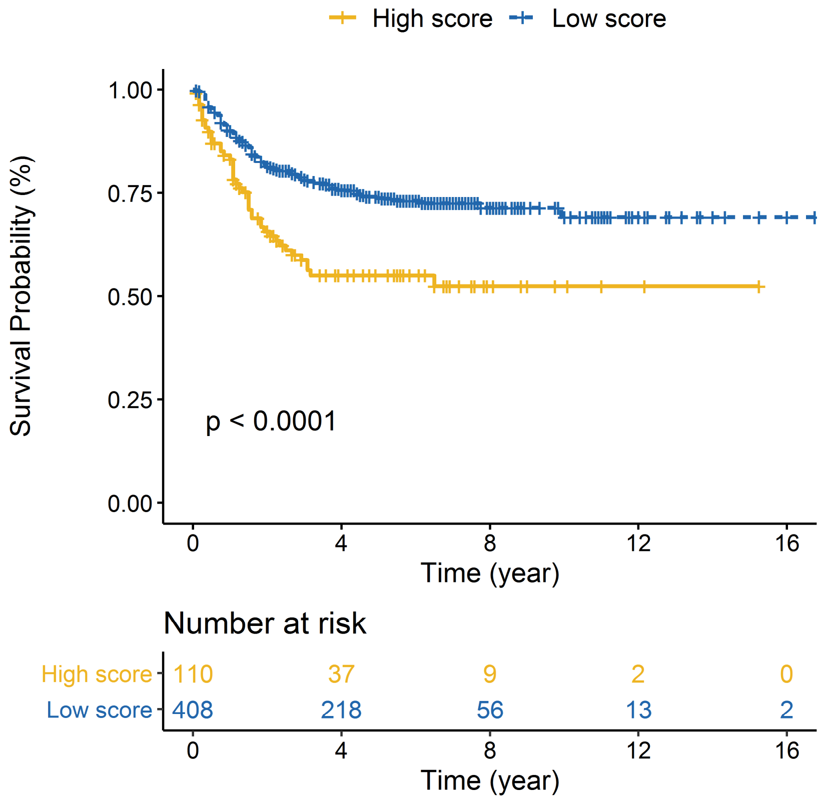

Figure S5. disease-free survival (DFS) analysis of immunoscore in GEO cohort. We divided the GEO CRC samples with disease-free survival information into low and high score group, using *survminer* package obtaining the optimal cut-off value. The result suggested that significant difference between low and high immunoscore group.

Supplementary Tables

Table S1. GSEA enrichment in $TP53^{WT}$ CRCs.

NAME	SIZE	ES	NES	NOM *p*-Val	FDR *q*-Val	FWER *p*-Val	RANK At MAX	LEADING EDGE
KEGG_INTESTINAL_IMMUNE_NETWORK_FOR_IGA_PRODUCTION	44	-0.70353	-2.31151	0	0	0	1984	tags=45%, list=9%, signal=50%
KEGG_DRUG_METABOLISM_CYTOCHROME_P450	58	-0.63391	-2.16678	0	0	0	2760	tags=41%, list=13%, signal=47%
KEGG_HEMATOPOIETIC_CELL_LINEAGE	83	-0.59057	-2.1537	0	0	0	3373	tags=39%, list=16%, signal=45%
KEGG_CYTOKINE_CYTOKINE_RECEPTOR_INTERACTION	246	-0.48829	-2.067	0	3.56E-04	0.001	3926	tags=37%, list=18%, signal=44%
KEGG_METABOLISM_OF_XENOBIOTICS_BY_CYTOCHROME_P450	55	-0.60057	-2.04912	0	2.85E-04	0.001	2760	tags=42%, list=13%, signal=48%
KEGG_GRAFT_VERSUS_HOST_DISEASE	35	-0.65688	-2.0394	0	4.56E-04	0.002	1984	tags=43%, list=9%, signal=47%
KEGG_RETINOL_METABOLISM	46	-0.63174	-2.03775	0	3.91E-04	0.002	2760	tags=41%, list=13%, signal=47%
KEGG_LEISHMANIA_INFECTION	67	-0.57511	-2.03133	0	3.42E-04	0.002	4569	tags=49%, list=21%, signal=62%
KEGG_PORPHYRIN_AND_CHLOROPHYLL_METABOLISM	29	-0.6758	-2.01935	0	3.04E-04	0.002	2500	tags=38%, list=12%, signal=43%
KEGG_APOPTOSIS	86	-0.53917	-1.97928	0	2.74E-04	0.002	3612	tags=41%, list=17%, signal=49%
KEGG_STEROID_HORMONE_BIOSYNTHESIS	41	-0.62604	-1.97467	0	3.70E-04	0.003	2365	tags=37%, list=11%, signal=41%
KEGG_STARCH_AND_SUCROSE_METABOLISM	36	-0.62028	-1.95278	0	4.58E-04	0.004	5063	tags=61%, list=23%, signal=80%
KEGG_P53_SIGNALING_PATHWAY	66	-0.55012	-1.94411	0	5.24E-04	0.005	3041	tags=36%, list=14%, signal=42%
KEGG_TOLL_LIKE_RECEPTOR_SIGNALING_PATHWAY	96	-0.51639	-1.92565	0	0.001373	0.014	1733	tags=25%, list=8%, signal=27%
KEGG_VALINE_LEUCINE_AND_ISOLEUCINE_DEGRADATION	44	-0.58782	-1.91123	0	0.001903	0.02	5367	tags=68%, list=25%, signal=90%
KEGG_PRION_DISEASES	34	-0.61209	-1.89546	0	0.002624	0.03	3082	tags=44%, list=14%, signal=51%
KEGG_TRYPTOPHAN_METABOLISM	39	-0.58856	-1.88988	0	0.00286	0.035	3946	tags=44%, list=18%, signal=53%
KEGG_SPHINGOLIPID_METABOLISM	34	-0.59519	-1.85496	0	0.004715	0.061	2004	tags=32%, list=9%, signal=36%
KEGG_NATURAL_KILLER_CELL_MEDIATED_CYTOTOXICITY	127	-0.45224	-1.76152	0	0.012228	0.17	4671	tags=41%, list=22%, signal=52%
KEGG_DRUG_METABOLISM_OTHER_ENZYMES	37	-0.55585	-1.74709	0	0.013445	0.208	4312	tags=43%, list=20%, signal=54%
KEGG_VIRAL_MYOCARDITIS	65	-0.47679	-1.68384	0	0.021266	0.361	4831	tags=38%, list=22%, signal=49%
KEGG_CHEMOKINE_SIGNALING_PATHWAY	180	-0.41253	-1.68352	0	0.020626	0.361	4339	tags=36%, list=20%, signal=45%
KEGG_PATHWAYS_IN_CANCER	319	-0.33601	-1.43604	0	0.101886	0.988	4023	tags=27%, list=19%, signal=33%
KEGG_MAPK_SIGNALING_PATHWAY	254	-0.35136	-1.47549	0.001279	0.089827	0.965	4042	tags=27%, list=19%, signal=33%
KEGG_T_CELL_RECEPTOR_SIGNALING_PATHWAY	107	-0.42991	-1.64603	0.00142	0.026711	0.49	2936	tags=26%, list=14%, signal=30%
KEGG_COMPLEMENT_AND_COAGULATION_CASCADES	67	-0.49834	-1.73179	0.001493	0.014709	0.249	5138	tags=45%, list=24%, signal=59%
KEGG_NOD_LIKE_RECEPTOR_SIGNALING_PATHWAY	60	-0.46986	-1.63961	0.001511	0.028273	0.52	4163	tags=37%, list=19%, signal=45%
KEGG_JAK_STAT_SIGNALING_PATHWAY	149	-0.39972	-1.5928	0.002717	0.043939	0.695	2776	tags=24%, list=13%, signal=28%
KEGG_FATTY_ACID_METABOLISM	40	-0.56887	-1.80955	0.003115	0.007835	0.104	5636	tags=73%, list=26%, signal=98%
KEGG_LINOLEIC_ACID_METABOLISM	23	-0.62915	-1.79603	0.003442	0.008586	0.119	2256	tags=39%, list=10%, signal=44%
KEGG_ALLOGRAFT_REJECTION	33	-0.56656	-1.74241	0.00463	0.013081	0.221	1984	tags=33%, list=9%, signal=37%

KEGG_TYPE_I_DIABETES_MELLITUS	39	-0.54931	-1.74275	0.004831	0.013498	0.219	1984	tags=36%, list=9%, signal=39%
KEGG_BUTANOATE_METABOLISM	33	-0.56786	-1.70488	0.00495	0.01902	0.317	3224	tags=39%, list=15%, signal=46%
KEGG_GLYCOSAMINOGLYCAN_DEGRADATION	20	-0.64509	-1.75918	0.005102	0.011977	0.175	2673	tags=25%, list=12%, signal=28%
KEGG_PATHOGENIC_ESCHERICHIA_COLI_INFECTION	51	-0.5028	-1.69951	0.006279	0.019431	0.33	1733	tags=20%, list=8%, signal=21%
KEGG_PANTOTHENATE_AND_COA_BIOSYNTHESIS	16	-0.68205	-1.75052	0.006757	0.013309	0.201	2503	tags=44%, list=12%, signal=49%
KEGG_AMINO_SUGAR_AND_NUCLEOTIDE_SUGAR_METABOLISM	42	-0.51033	-1.65038	0.007553	0.026344	0.474	3207	tags=33%, list=15%, signal=39%
KEGG_FC_GAMMA_R_MEDIATED_PHAGOCYTOSIS	93	-0.40422	-1.49482	0.008608	0.081697	0.93	4167	tags=35%, list=19%, signal=44%
KEGG_GLYCOLYSIS_GLUCONEOGENESIS	60	-0.44283	-1.55065	0.009217	0.060194	0.823	5745	tags=47%, list=27%, signal=63%
KEGG_SYSTEMIC_LUPUS_ERYTHEMATOSUS	96	-0.427	-1.57381	0.011611	0.051873	0.766	4856	tags=35%, list=22%, signal=45%
KEGG_LEUKOCYTE_TRANSENDOTHELIAL_MIGRATION	113	-0.38544	-1.47143	0.012931	0.089438	0.969	3348	tags=27%, list=15%, signal=31%
KEGG_NITROGEN_METABOLISM	22	-0.59299	-1.65835	0.013311	0.025201	0.443	3148	tags=41%, list=15%, signal=48%
KEGG_CYTOSOLIC_DNA_SENSING_PATHWAY	51	-0.46826	-1.55025	0.015408	0.058888	0.825	2583	tags=29%, list=12%, signal=33%
KEGG_COLORECTAL_CANCER	62	-0.43271	-1.50614	0.015432	0.077064	0.919	4477	tags=39%, list=21%, signal=49%
KEGG_PHOSPHATIDYLINOSITOL_SIGNALING_SYSTEM	74	-0.41566	-1.49709	0.017673	0.081724	0.926	3865	tags=34%, list=18%, signal=41%
KEGG_O_GLYCAN_BIOSYNTHESIS	26	-0.56149	-1.63378	0.017699	0.029174	0.548	1194	tags=31%, list=6%, signal=33%
KEGG_B_CELL_RECEPTOR_SIGNALING_PATHWAY	74	-0.41108	-1.46872	0.024691	0.088127	0.971	4450	tags=36%, list=21%, signal=46%
KEGG_ALDOSTERONE_REGULATED_SODIUM_REABSORPTION	41	-0.49531	-1.55492	0.02589	0.058964	0.804	3041	tags=29%, list=14%, signal=34%
KEGG_GLYCEROPHOSPHOLIPID_METABOLISM	70	-0.4031	-1.41315	0.028919	0.113837	0.992	3506	tags=29%, list=16%, signal=34%
KEGG_AMYOTROPHIC_LATERAL_SCLEROSIS_ALS	51	-0.44706	-1.4939	0.030395	0.080688	0.932	4671	tags=37%, list=22%, signal=47%
KEGG_ARACHIDONIC_ACID_METABOLISM	50	-0.44507	-1.47353	0.030844	0.08949	0.965	1862	tags=28%, list=9%, signal=31%
KEGG_RENAL_CELL_CARCINOMA	69	-0.40547	-1.4426	0.031818	0.100232	0.985	4879	tags=35%, list=23%, signal=45%
KEGG_FC_EPSILON_RI_SIGNALING_PATHWAY	73	-0.40819	-1.46988	0.034691	0.088885	0.97	4163	tags=34%, list=19%, signal=42%
KEGG_BETA_ALANINE_METABOLISM	22	-0.54309	-1.5209	0.035413	0.069876	0.897	4149	tags=55%, list=19%, signal=67%
KEGG_ETHER_LIPID_METABOLISM	28	-0.50248	-1.48352	0.036244	0.08573	0.953	3354	tags=39%, list=15%, signal=46%
KEGG_TYROSINE_METABOLISM	42	-0.45137	-1.46472	0.039185	0.08948	0.974	2761	tags=29%, list=13%, signal=33%
KEGG_GLYCOSPHINGOLIPID_BIOSYNTHESIS_LACTO_AND_NEOLACTO_SERIES	26	-0.52135	-1.52852	0.042414	0.068724	0.882	4649	tags=50%, list=21%, signal=64%
KEGG_INOSITOL_PHOSPHATE_METABOLISM	54	-0.42944	-1.44358	0.043741	0.101237	0.985	3865	tags=35%, list=18%, signal=43%
KEGG_SMALL_CELL_LUNG_CANCER	84	-0.37383	-1.38164	0.043924	0.13738	0.998	4155	tags=32%, list=19%, signal=40%
KEGG_PRIMARY_IMMUNODEFICIENCY	35	-0.4659	-1.43641	0.044925	0.10327	0.988	2875	tags=31%, list=13%, signal=36%
KEGG_CHRONIC_MYELOID_LEUKEMIA	72	-0.39265	-1.40217	0.045455	0.120121	0.994	4163	tags=31%, list=19%, signal=38%

Table S2. GSEA enrichment in $TP53^{M}$ CRCs.

NAME	SIZE	ES	NES	NOM *p*-Val	FDR *q*-Val	FWER *p*-Val	RANK AT MAX	LEADING EDGE
KEGG_DNA_REPLICATION	36	0.545324	1.839467	0	0.030854	0.08	5759	Tags = 50%, list = 27%, signal = 68%
KEGG_HOMOLOGOUS_RECOMBINATION	28	0.53746	1.748391	0.008264	0.040201	0.191	4124	Tags = 43%, list = 19%, signal = 53%

Table S3. Differentially expressed genes between $TP53^{M}$ and $TP53^{WT}$ in CRCs.

Symbol	LogFC	AveExpr	*t*	*p*.Value	adj.*p*.Val	Symbol	LogFC	AveExpr	*t*	*p*.Value	adj.*p*.Val
TCN1	1.269601	6.369232	5.338203	1.69E-07	2.36E-05	RARRES1	0.434333	7.20121	2.769761	0.005908	0.042109
AGR3	1.217781	8.654876	5.252907	2.60E-07	3.20E-05	CCDC68	0.432931	6.097877	4.136167	4.42E-05	0.001408
CLCA1	1.147779	8.039989	3.525085	0.000479	0.007781	CARD6	0.431502	5.556473	4.627327	5.22E-06	0.00031
REG1A	1.118144	8.124658	3.449046	0.000631	0.009462	CHP1	0.430562	8.620064	7.168027	4.51E-12	5.20E-09
FCGBP	1.095105	8.12191	4.256419	2.67E-05	0.000978	LPIN1	0.424874	6.459636	5.971193	5.76E-09	1.69E-06
REG4	1.003115	7.346541	3.809755	0.000164	0.003654	MUC1	0.42368	7.623829	3.509885	0.000507	0.008091
MUC2	0.97981	8.82244	4.001248	7.69E-05	0.002137	OSR2	0.421729	4.665589	3.695663	0.000254	0.004983
SPINK4	0.973467	8.652496	3.510941	0.000505	0.008077	C11orf92	0.419304	2.965485	5.335486	1.71E-07	2.38E-05
L1TD1	0.959767	5.456801	5.104959	5.43E-07	5.43E-05	CYP2C18	0.417519	4.674835	4.127898	4.58E-05	0.001436
PIGR	0.955576	9.949745	4.47657	1.03E-05	0.000504	PAX8-AS1	0.417381	6.164945	2.886126	0.004141	0.033656
REG1B	0.925315	6.645757	3.082329	0.002216	0.021933	CD200	0.417348	5.625558	4.283196	2.38E-05	0.000904
DEFA5	0.796548	6.246102	2.707237	0.007116	0.047767	RP11-363E7.4	0.417145	4.440831	4.556308	7.19E-06	0.000397
SLITRK6	0.795226	4.212359	5.020361	8.21E-07	7.41E-05	CAPN9	0.415791	4.899448	3.758684	0.0002	0.004224
RETNLB	0.783671	6.17199	3.99265	7.96E-05	0.002197	TMEM45A	0.415088	6.463684	3.188159	0.00156	0.017462
HEPACAM2	0.780764	6.022123	3.193948	0.00153	0.017241	TLR4	0.413272	5.074302	4.530137	8.09E-06	0.000426
CTSE	0.773958	6.685974	3.868154	0.000131	0.003162	TRIM16	0.410211	6.971087	4.61654	5.48E-06	0.000318
DUSP4	0.741596	6.113443	5.681021	2.81E-08	5.59E-06	C12orf57	0.408571	9.458935	5.83677	1.21E-08	2.91E-06
ZIC2	0.725346	4.750312	3.272535	0.001172	0.014448	FGFBP1	0.407954	6.703023	3.614798	0.000344	0.006095
FAM169A	0.703682	4.992292	5.466019	8.74E-08	1.41E-05	GIF	0.406924	3.820008	4.040792	6.55E-05	0.001895
RPL22L1	0.677375	9.477708	6.598473	1.53E-10	9.75E-08	PKIB	0.406825	5.179295	2.859441	0.004497	0.035401
PLA2G4A	0.673671	5.416364	3.569121	0.000408	0.006869	FOXD1	0.406556	4.777472	2.833809	0.004865	0.036994
SLC6A14	0.669019	5.605909	3.311788	0.001023	0.013081	LIMA1	0.405917	7.784509	4.92777	1.28E-06	0.000111
LYZ	0.667556	9.770075	3.927452	0.000103	0.002659	FDXR	0.405524	5.828777	5.131348	4.77E-07	5.09E-05
CA2	0.666632	8.099356	3.183645	0.001584	0.017611	VNN1	0.405412	4.476923	3.357997	0.000871	0.01179
NRN1	0.6659	5.640258	4.316726	2.06E-05	0.000818	TLR3	0.403601	4.544338	5.194734	3.48E-07	4.05E-05
HOXC6	0.657121	5.526046	3.325367	0.000976	0.012651	PCSK1	0.403013	5.521496	1.737165	0.083234	0.23018
PLK2	0.646179	6.872774	6.279585	1.00E-09	3.88E-07	RASGRP1	0.401207	4.779856	4.466616	1.07E-05	0.00052
SAMD5	0.640757	5.790339	4.459276	1.11E-05	0.000526	CRIP1	0.400858	8.717976	3.105312	0.002055	0.020807
ITLN1	0.63573	7.724173	2.151737	0.032099	0.125288	RLN2	-0.40267	4.018778	-4.54755	7.48E-06	0.000403
CXCL13	0.630136	5.43476	3.109828	0.002025	0.020644	TP53RK	-0.40271	7.446554	-6.20458	1.54E-09	5.57E-07
HYAL1	0.618335	5.775861	4.45894	1.11E-05	0.000526	FAM217B	-0.40515	7.185665	-4.32978	1.95E-05	0.000786
HSPA4L	0.61726	4.464035	4.481655	1.00E-05	0.000498	TRIB3	-0.40547	7.128013	-5.05506	6.94E-07	6.68E-05
CFI	0.616197	6.588913	5.121104	5.02E-07	5.16E-05	CYP4F2	-0.40574	5.843152	-3.43052	0.000674	0.00983
ALDH1A1	0.61307	8.016419	3.637419	0.000317	0.005781	SLC26A3	-0.40643	6.356646	-1.80176	0.07244	0.211612
LCN2	0.610664	11.05236	3.563172	0.000417	0.006994	SLMO2	-0.40647	8.36702	-5.9376	6.95E-09	1.93E-06

SLCO1B3	0.609407	5.129574	2.71825	0.006888	0.046686
RNF125	0.605769	5.376517	5.88919	9.08E-09	2.31E-06
CLDN2	0.602566	6.584847	3.484787	0.000555	0.008551
FAS	0.598622	6.522679	6.279471	1.00E-09	3.88E-07
CPS1	0.596691	3.955464	3.248374	0.001273	0.015225
SCG5	0.593812	5.960343	4.26812	2.54E-05	0.000946
ADTRP	0.590459	5.703308	4.03665	6.66E-05	0.00192
MLPH	0.589999	8.497168	4.567472	6.84E-06	0.000382
PLA2G2A	0.587976	9.499693	2.442132	0.015093	0.07723
SPATA18	0.58713	4.669889	10.41009	2.67E-22	5.79E-18
TRIM22	0.582621	8.151523	5.009753	8.65E-07	7.77E-05
IL1R2	0.582506	6.553516	4.464678	1.08E-05	0.00052
PLAC8	0.577719	9.200014	2.90989	0.003846	0.032044
ST6GALNAC1	0.568108	8.898053	2.918016	0.003749	0.031519
SBSPON	0.564092	4.507489	4.395887	1.46E-05	0.000645
FFAR4	0.563162	4.644271	4.306244	2.16E-05	0.000834
LINC00261	0.555265	6.741698	3.127799	0.001908	0.019881
COLCA2	0.549437	5.54353	3.576323	0.000397	0.006736
EGLN3	0.541692	6.37259	5.270688	2.38E-07	2.99E-05
TFF1	0.534207	10.24866	2.486143	0.013377	0.071334
RPS27L	0.526132	8.046896	9.699066	7.26E-20	7.86E-16
MRAP2	0.520678	6.200264	4.115009	4.83E-05	0.001483
ASRGL1	0.518983	7.326307	4.838359	1.96E-06	0.00015
DUOX2	0.518406	7.74777	2.192342	0.02901	0.117094
AGR2	0.517969	10.40666	4.01251	7.34E-05	0.002065
SDR16C5	0.513119	6.629785	2.626388	0.009007	0.055617
HSD17B2	0.510931	6.460643	2.95698	0.003317	0.02888
CXCL11	0.510708	5.346254	2.409273	0.016498	0.081793
SEMG1	0.510278	3.423634	4.031616	6.80E-05	0.001957
TNFSF9	0.499443	5.121148	5.116727	5.13E-07	5.21E-05
LOC100505501	0.496974	4.500985	5.490558	7.69E-08	1.29E-05
SGPP2	0.491955	6.673975	4.524682	8.29E-06	0.000431
REG3A	0.4914	5.83741	2.804364	0.005322	0.039225
TSPAN1	0.490636	10.19203	4.014309	7.29E-05	0.002056
ZMAT3	0.490544	6.385927	7.312959	1.78E-12	2.57E-09
TNFRSF11A	0.48893	6.723054	4.197566	3.42E-05	0.001176
GNAI1	0.488011	5.756917	4.271777	2.50E-05	0.000937
PTGER2	0.487547	5.380696	4.627356	5.22E-06	0.00031

ASXL1	-0.41054	6.996167	-7.40912	9.54E-13	1.48E-09
LDLRAD3	-0.41451	6.750412	-4.20634	3.30E-05	0.001154
RNF43	-0.41908	9.380016	-3.85992	0.000135	0.003219
PROSER1	-0.42016	8.049286	-5.46325	8.87E-08	1.42E-05
GGH	-0.42103	9.802513	-3.9008	0.000115	0.002867
VAV3	-0.42315	7.614138	-2.75678	0.006142	0.043183
Y16709	-0.4235	11.6614	-4.2955	2.26E-05	0.000864
SYNE4	-0.42429	5.995705	-3.81231	0.000163	0.003636
ACOT8	-0.42458	6.415288	-6.47933	3.11E-10	1.57E-07
PCP4	-0.42773	5.048334	-2.39564	0.017114	0.083669
DPM1	-0.4302	10.23169	-7.22812	3.07E-12	3.92E-09
PSMA7	-0.43362	9.84254	-7.95681	2.45E-14	7.28E-11
C8orf33	-0.43694	7.999023	-5.83815	1.20E-08	2.91E-06
MRGBP	-0.43872	6.060199	-7.06864	8.47E-12	7.98E-09
PLTP	-0.43932	7.712448	-3.84698	0.000142	0.003337
DNTTIP1	-0.43942	8.78904	-6.48053	3.09E-10	1.57E-07
TCFL5	-0.44161	6.514549	-6.20913	1.50E-09	5.52E-07
PRSS33	-0.44449	5.267091	-3.20427	0.001478	0.016898
MYBL2	-0.44806	7.390008	-6.01175	4.60E-09	1.40E-06
CTNNBL1	-0.45321	8.159247	-7.88234	4.08E-14	9.81E-11
MPP1	-0.45363	7.50899	-4.60944	5.66E-06	0.000323
PHACTR3	-0.45815	3.782344	-3.94555	9.61E-05	0.002521
DDX27	-0.46118	8.501022	-7.9433	2.69E-14	7.28E-11
AP000525.9	-0.46254	5.117536	-4.50909	8.88E-06	0.000451
PLCB4	-0.46764	8.657532	-2.62763	0.008975	0.05553
PLAGL2	-0.46904	6.942156	-5.2929	2.12E-07	2.82E-05
ATP9A	-0.47583	7.843547	-5.38498	1.33E-07	1.94E-05
LOC729680	-0.48254	8.174807	-5.26728	2.42E-07	3.03E-05
CYP4F3	-0.48696	5.71196	-3.72569	0.000227	0.004617
CELP	-0.49118	6.183829	-2.90242	0.003937	0.032519
WASF3	-0.4937	5.225273	-3.28595	0.001119	0.013948
ASCL2	-0.49783	6.531319	-3.94933	9.47E-05	0.002495
NELFCD	-0.50065	9.69424	-7.71612	1.25E-13	2.54E-10
WIF1	-0.50096	3.725541	-2.81641	0.00513	0.038225
SCRN1	-0.50707	7.292297	-4.30737	2.15E-05	0.000831
FZD10	-0.50896	4.498966	-2.41917	0.016063	0.080463
MUC12	-0.51043	6.99585	-2.7002	0.007265	0.048467
EPDR1	-0.51236	8.064563	-3.57653	0.000397	0.006736

DMBT1	0.487508	8.706843	2.234789	0.026059	0.109087
EIF5A	0.484965	9.004405	4.130285	4.53E-05	0.001426
NR3C2	0.481446	7.04933	3.501271	0.000523	0.00825
MB	0.478914	5.529173	4.46495	1.08E-05	0.00052
HINT3	0.477332	5.523164	3.446623	0.000636	0.009501
SLC28A3	0.47502	4.366214	5.281109	2.26E-07	2.89E-05
RP11-38P22.2	0.472806	4.313585	4.080023	5.58E-05	0.001668
KLK11	0.47181	6.268693	3.910383	0.000111	0.002804
TM4SF4	0.471567	4.983154	3.549565	0.000438	0.007246
CYTIP	0.469524	5.93759	4.380087	1.57E-05	0.000681
NAT1	0.460697	7.682351	5.166091	4.01E-07	4.47E-05
RAB27B	0.459911	4.656846	5.646108	3.39E-08	6.38E-06
ADH6	0.455471	4.762203	4.361159	1.70E-05	0.000709
ARSJ	0.453229	4.634069	5.219986	3.07E-07	3.67E-05
LCN15	0.445305	6.368153	2.319486	0.020941	0.095169
DDB2	0.445016	6.811937	8.181533	5.19E-15	2.81E-11
CDKN1A	0.443645	8.795436	6.313983	8.21E-10	3.35E-07
SYTL1	0.442651	6.425098	4.260639	2.62E-05	0.000969
ARRDC4	0.44036	7.419633	4.721914	3.38E-06	0.000225
CASP1	0.440041	8.223464	3.804093	0.000168	0.003704
FUT8	0.439178	7.189515	5.31976	1.85E-07	2.54E-05
CFD	0.43714	7.292034	3.34676	0.000906	0.012062
DEFA6	0.436138	7.311844	1.750981	0.080822	0.226357
ANG	0.434936	7.996411	4.324014	2.00E-05	0.0008
TMEM71	0.434833	4.867747	3.508746	0.000509	0.008106
TMEM263	0.434728	8.221797	5.449251	9.53E-08	1.47E-05
BIRC3	0.434581	6.36247	5.109404	5.31E-07	5.35E-05
TTI1	-0.51542	7.323327	-8.08603	1.01E-14	4.36E-11
TPX2	-0.51801	9.296127	-6.50189	2.72E-10	1.51E-07
PMEPA1	-0.52045	8.499733	-5.11163	5.26E-07	5.32E-05
PRAP1	-0.52264	8.081154	-3.61523	0.000344	0.00609
PPP1R3D	-0.52658	6.084909	-7.71199	1.29E-13	2.54E-10
FOXQ1	-0.53711	9.773826	-3.9308	0.000102	0.002635
CPNE1	-0.54644	8.709606	-6.71282	7.67E-11	5.13E-08
RBP2	-0.55034	5.704719	-4.55447	7.25E-06	0.0004
CTSV	-0.5606	7.404533	-5.9194	7.68E-09	2.03E-06
GPR143	-0.58369	5.742498	-4.86976	1.69E-06	0.000136
CKMT2	-0.58451	5.30282	-3.3661	0.000847	0.011575
LAPTM4B	-0.59451	9.973948	-5.317	1.88E-07	2.54E-05
QPRT	-0.60723	7.653147	-4.02642	6.94E-05	0.001986
CEL	-0.60954	7.034082	-2.28244	0.023061	0.101191
UCA1	-0.61236	6.783806	-3.51976	0.000489	0.007862
KIAA0226L	-0.61513	8.597197	-3.4772	0.00057	0.008751
UBE2C	-0.6158	9.904635	-7.60913	2.56E-13	4.62E-10
PIPOX	-0.62114	4.81597	-4.68337	4.04E-06	0.000258
BEX2	-0.623	5.832191	-3.84032	0.000146	0.003394
COL9A3	-0.69521	6.067645	-5.03455	7.67E-07	7.15E-05
SLC35D3	-0.75712	5.49688	-3.63655	0.000318	0.005787
PRAC1	-0.76746	7.010654	-2.33107	0.020314	0.093459
GNG4	-0.78555	5.313431	-6.5472	2.08E-10	1.22E-07
MAP7D2	-0.89302	5.173364	-4.74615	3.02E-06	0.000205
EREG	-0.90448	6.128234	-5.93859	6.91E-09	1.93E-06
KRT23	-1.23793	7.40819	-5.1355	4.67E-07	5.03E-05

Table S4. Differentially expressed genes between subgroups based on proportion of immune cells in CRC.

Symbol	LogFC	AveExpr	*t*	*p*.Value	adj.*p*.Val	Symbol	LogFC	AveExpr	*t*	*p*.Value	adj.*p*.Val
CLCA1	2.168179	7.980473	6.861179	2.26E-11	4.45E-09	CHN1	-0.45599	6.91166	-6.08499	2.49E-09	2.75E-07
IGJ	2.06133	10.00636	10.62714	1.08E-23	5.82E-20	MYH10	-0.45753	5.951414	-6.32563	6.08E-10	7.88E-08
UGT2B17	1.705958	5.828082	6.12434	1.98E-09	2.25E-07	MSRB3	-0.45812	5.915235	-5.72886	1.85E-08	1.53E-06
ITLN1	1.680523	7.571623	5.964412	4.96E-09	5.00E-07	C10orf10	-0.45854	7.149695	-5.66619	2.60E-08	2.04E-06
ZG16	1.633529	7.383166	6.258261	9.06E-10	1.13E-07	UBE2E2	-0.45934	6.609889	-4.25418	2.55E-05	0.000668
MS4A12	1.457647	5.603321	5.628557	3.20E-08	2.41E-06	EFEMP2	-0.45992	7.416823	-4.86403	1.59E-06	6.95E-05
CLCA4	1.441808	5.479832	5.697808	2.19E-08	1.77E-06	NEXN	-0.46015	5.579004	-3.37638	0.000798	0.010992
CEACAM7	1.433024	7.773292	5.807085	1.20E-08	1.07E-06	SERPINH1	-0.46224	9.340522	-6.36655	4.76E-10	6.36E-08
SI	1.40316	4.769757	5.949863	5.39E-09	5.38E-07	TMEM158	-0.46264	7.48669	-4.83116	1.86E-06	7.91E-05
HEPACAM2	1.300983	5.929346	5.614053	3.46E-08	2.57E-06	C11orf96	-0.46292	9.220103	-4.21642	3.00E-05	0.000766
SPINK4	1.24075	8.676055	4.458448	1.04E-05	0.000324	HMCN1	-0.46423	4.617294	-4.41255	1.28E-05	0.000383
ADAMDEC1	1.22299	8.111762	7.539398	2.61E-13	9.92E-11	MCAM	-0.46553	7.436492	-5.99826	4.09E-09	4.18E-07
FCGBP	1.205576	8.110353	4.707576	3.34E-06	0.000127	PLEK	-0.46945	6.601188	-5.70803	2.07E-08	1.69E-06
ADH1C	1.201454	6.263778	5.686505	2.33E-08	1.86E-06	GUCY1A3	-0.47001	6.194959	-4.27306	2.35E-05	0.000627
TNFRSF17	1.142991	4.837849	7.877697	2.51E-14	1.26E-11	PKD2	-0.47268	6.437105	-4.82067	1.96E-06	8.23E-05
OLFM4	1.080172	10.17178	3.351538	0.000871	0.01182	BCL6	-0.47272	6.295671	-7.86366	2.77E-14	1.36E-11
AKR1B10	1.074759	6.780632	5.770289	1.47E-08	1.26E-06	MFAP5	-0.47297	5.015791	-3.12759	0.001876	0.021522
DUOX2	0.984501	7.473184	4.316977	1.95E-05	0.000537	ADAMTS5	-0.47399	4.879779	-6.90705	1.69E-11	3.60E-09
PLAC8	0.96645	9.180657	5.086416	5.36E-07	2.70E-05	AOC3	-0.47431	6.454179	-3.60984	0.000341	0.005535
REG4	0.959474	7.435246	3.673743	0.000268	0.004561	PLXDC2	-0.4752	5.37404	-4.93133	1.15E-06	5.31E-05
IGLL5	0.9479	8.776331	6.663746	7.79E-11	1.32E-08	PLAUR	-0.477	8.039195	-6.33324	5.81E-10	7.58E-08
ADTRP	0.94435	5.580979	7.015827	8.41E-12	1.96E-09	CLMP	-0.47795	5.651944	-4.72326	3.10E-06	0.00012
CA2	0.930706	8.060403	4.532441	7.48E-06	0.000248	HSPA6	-0.48336	5.814782	-5.14606	3.98E-07	2.13E-05
CA4	0.919263	5.750173	5.674073	2.50E-08	1.96E-06	GUCY1B3	-0.48411	6.230944	-5.44395	8.57E-08	5.56E-06
GCG	0.906447	3.851145	5.716421	1.98E-08	1.63E-06	ARMCX2	-0.48441	6.693687	-4.33218	1.82E-05	0.000515
PLA2G2A	0.901631	9.64481	3.94592	9.22E-05	0.001915	CCDC80	-0.48459	5.712352	-4.52732	7.65E-06	0.000252
DHRS9	0.897509	6.614024	5.701873	2.14E-08	1.74E-06	ZNF521	-0.48495	4.512763	-5.14769	3.94E-07	2.11E-05
IGHM	0.896282	7.587264	7.153334	3.44E-12	8.98E-10	SELM	-0.48507	8.352052	-4.96643	9.69E-07	4.59E-05
PIGR	0.894439	9.844754	4.3559	1.64E-05	0.000471	PDGFC	-0.48588	6.514497	-4.37179	1.53E-05	0.000443
MUC2	0.890907	8.887077	3.603605	0.000349	0.005632	LUM	-0.48725	8.601387	-4.61364	5.16E-06	0.000181
IGLL3P	0.883637	9.221702	7.210868	2.36E-12	6.63E-10	ANGPTL2	-0.48762	6.487916	-5.61688	3.40E-08	2.54E-06
PRAC1	0.861368	6.955187	2.705894	0.00707	0.056762	SLIT2	-0.48989	5.256438	-3.97442	8.21E-05	0.001742
NXPE4	0.846872	6.709422	4.726055	3.06E-06	0.000119	MEIS1	-0.49009	5.53843	-4.63277	4.73E-06	0.000169
C10orf99	0.839403	8.511049	3.651827	0.000291	0.004864	SLC16A10	-0.49094	4.166594	-8.09982	5.18E-15	3.12E-12
SLC26A3	0.82447	6.143063	3.699869	0.000242	0.004215	CRIP2	-0.49101	6.829079	-6.39355	4.05E-10	5.52E-08

KLRB1	0.809093	6.04437	8.657135	8.62E-17	7.46E-14	MLLT11	-0.49122	6.784981	-5.18772	3.22E-07	1.79E-05
MZB1	0.792798	6.532629	6.808727	3.15E-11	6.03E-09	CST1	-0.49389	6.936876	-2.44484	0.014873	0.093398
GUCA2A	0.782375	6.724013	4.867314	1.57E-06	6.87E-05	FCGR2B	-0.49426	5.55378	-4.11774	4.55E-05	0.001076
IGK	0.762328	7.401994	6.122633	2.00E-09	2.26E-07	ZCCHC24	-0.49526	7.473261	-4.85441	1.67E-06	7.19E-05
CLDN8	0.757986	3.669609	4.706964	3.35E-06	0.000127	CD93	-0.49578	7.372293	-7.09872	4.91E-12	1.21E-09
SPINK5	0.732122	6.184407	5.162867	3.65E-07	1.97E-05	ELTD1	-0.50143	6.343053	-5.90983	6.75E-09	6.62E-07
IGLV1-44	0.730793	7.164157	6.871803	2.11E-11	4.24E-09	FPR2	-0.50204	4.639001	-7.58039	1.97E-13	8.38E-11
LRRC19	0.72135	5.832321	4.329425	1.84E-05	0.000519	AK021804	-0.50279	4.817562	-3.91187	0.000106	0.002133
VSIG2	0.717074	6.41086	6.477521	2.44E-10	3.57E-08	FIBIN	-0.5042	5.478587	-5.47136	7.41E-08	4.96E-06
CHP2	0.716282	6.387688	4.786238	2.31E-06	9.28E-05	GLIS2	-0.50437	6.283055	-5.64538	2.92E-08	2.25E-06
RETNLB	0.693005	6.141621	3.689338	0.000252	0.004342	GPNMB	-0.50442	6.519792	-4.23018	2.83E-05	0.000727
CXCL14	0.688304	7.600141	4.651572	4.33E-06	0.000156	APOD	-0.50565	6.809785	-3.01676	0.002699	0.028347
HSD17B2	0.686174	6.367727	3.997565	7.47E-05	0.001625	IL1RN	-0.50899	4.960362	-9.34788	4.15E-19	5.79E-16
TRBC1	0.681992	7.793808	7.071769	5.86E-12	1.38E-09	TIMP2	-0.51071	8.886353	-5.23757	2.50E-07	1.42E-05
DNASE1L3	0.667175	4.842545	9.344877	4.25E-19	5.79E-16	EMILIN1	-0.51122	6.878459	-5.15804	3.74E-07	2.02E-05
IGLC1	0.662883	7.91371	7.637115	1.34E-13	5.80E-11	CYR61	-0.5116	8.74018	-4.15059	3.96E-05	0.000973
IGLJ3	0.657917	6.906963	6.670014	7.50E-11	1.29E-08	ITGBL1	-0.51279	4.318137	-5.48275	6.98E-08	4.72E-06
CASP1	0.653882	8.105755	6.067284	2.76E-09	3.01E-07	EPYC	-0.51356	3.4802	-2.82075	0.005002	0.044467
IDO1	0.64827	6.760913	4.82511	1.92E-06	8.09E-05	NUAK1	-0.51397	6.972483	-5.59586	3.81E-08	2.80E-06
XIST	0.646758	5.55044	2.538262	0.011475	0.078864	FCGR3B	-0.51411	6.219154	-4.71176	3.27E-06	0.000126
ARL14	0.644488	6.805873	4.688394	3.65E-06	0.000136	DPYSL3	-0.51679	5.710072	-5.79159	1.31E-08	1.14E-06
MEP1A	0.636435	8.220414	3.425255	0.00067	0.009525	NDUFA4L2	-0.51777	6.660577	-5.89916	7.17E-09	6.96E-07
NXPE1	0.610441	5.65505	4.809628	2.06E-06	8.55E-05	CTGF	-0.51853	9.48861	-4.77126	2.48E-06	9.87E-05
SULT1B1	0.609115	6.472257	5.19042	3.18E-07	1.77E-05	SYNC	-0.51903	4.620148	-4.73846	2.89E-06	0.000113
RNF186	0.606595	7.43968	4.779414	2.38E-06	9.55E-05	PRKCDBP	-0.52045	6.932353	-5.22538	2.66E-07	1.51E-05
CA1	0.601331	4.957087	5.639554	3.01E-08	2.29E-06	NID2	-0.52119	7.552209	-5.14353	4.03E-07	2.15E-05
CD8A	0.600279	6.28493	6.299043	7.12E-10	9.06E-08	HOXC6	-0.52149	5.789085	-2.54047	0.011404	0.078548
CCL28	0.599812	5.983548	5.298696	1.83E-07	1.08E-05	S1PR3	-0.52278	5.627023	-9.14115	2.11E-18	2.54E-15
BEST2	0.592625	4.869266	5.12823	4.35E-07	2.27E-05	MMP13	-0.52315	4.085849	-7.23912	1.96E-12	5.65E-10
GZMA	0.59243	6.068637	4.598181	5.54E-06	0.000192	PTRF	-0.52694	7.219762	-6.00157	4.02E-09	4.12E-07
SLC6A14	0.592278	5.474373	2.966079	0.003176	0.032035	THY1	-0.52695	8.11477	-5.71354	2.01E-08	1.65E-06
AGR3	0.591597	8.631695	2.465106	0.014068	0.09016	SDC2	-0.52765	6.963887	-5.90206	7.06E-09	6.88E-07
UGT2A3	0.587084	4.044609	3.670814	0.000271	0.004597	CDH11	-0.52886	6.496729	-5.1761	3.42E-07	1.88E-05
CD3D	0.580417	7.62578	8.084237	5.79E-15	3.39E-12	PLAU	-0.53171	7.615829	-5.41994	9.72E-08	6.16E-06
SCGB2A1	0.577077	4.243195	4.514838	8.10E-06	0.000264	CHI3L1	-0.5353	6.299641	-5.4458	8.49E-08	5.54E-06
IGKC	0.573048	6.77638	7.576549	2.03E-13	8.44E-11	OSM	-0.53583	5.054926	-9.41775	2.39E-19	4.13E-16
SOSTDC1	0.572993	5.776997	4.079856	5.33E-05	0.001223	NOX4	-0.53683	3.83374	-7.50643	3.27E-13	1.19E-10
CD38	0.570007	5.469241	6.57138	1.38E-10	2.18E-08	MIR100HG	-0.54069	4.165852	-4.11579	4.59E-05	0.001082

NR3C2	0.565826	6.934786	4.319254	1.93E-05	0.000536
SLITRK6	0.562661	4.219466	3.478171	0.000554	0.008158
FABP1	0.562088	8.168518	3.029241	0.002592	0.027531
ID1	0.562057	10.45824	3.998169	7.46E-05	0.001623
GCNT3	0.555505	8.476699	3.519046	0.000477	0.007178
CD177	0.552751	5.927165	4.123699	4.44E-05	0.001056
SELENBP1	0.551667	9.113696	3.937775	9.52E-05	0.001966
CD2	0.549891	6.735446	6.160784	1.60E-09	1.88E-07
NAT2	0.548452	6.878861	4.395011	1.38E-05	0.000407
AKR1C3	0.543801	9.370735	3.695022	0.000247	0.004273
ACE2	0.542036	6.594732	2.946053	0.003385	0.033441
SLAMF7	0.541998	5.491213	6.146242	1.75E-09	2.01E-07
DUOXA2	0.539238	6.903462	4.12248	4.46E-05	0.00106
KRT20	0.53144	10.13021	2.645815	0.008434	0.06415
LGALS2	0.526641	6.216936	4.265434	2.43E-05	0.000641
GPR128	0.523711	4.71028	3.57878	0.000383	0.006069
SLC26A2	0.522714	8.092148	3.082267	0.00218	0.024209
CXCL13	0.522645	5.655877	2.650308	0.008324	0.063539
HSD11B2	0.521941	7.80891	4.197029	3.26E-05	0.000821
GPA33	0.510402	9.429963	4.003485	7.30E-05	0.001593
CASP5	0.50896	5.956474	4.846897	1.73E-06	7.42E-05
ACSM3	0.507112	5.786357	4.401066	1.35E-05	0.000398
SAMD13	0.504288	6.4776	4.327559	1.86E-05	0.00052
CHGA	0.503449	4.899906	5.424704	9.48E-08	6.07E-06
LRRC31	0.502744	5.432689	3.718344	0.000226	0.003958
DMBT1	0.496759	8.67449	2.27831	0.023174	0.124868
RTP4	0.494478	6.442162	5.541672	5.10E-08	3.61E-06
SLC51B	0.490356	5.370558	4.693436	3.57E-06	0.000134
UGT2B15	0.489844	4.932706	4.890601	1.40E-06	6.24E-05
PCSK1	0.489453	5.408044	2.241021	0.02551	0.132793
PPP1R14D	0.484493	7.698981	3.741454	0.000207	0.003681
XDH	0.482582	6.49923	4.301128	2.08E-05	0.000567
LOC100293211	0.473704	4.44841	5.342089	1.46E-07	8.85E-06
CES3	0.473329	6.915004	6.448135	2.92E-10	4.21E-08
KLF4	0.46744	8.450087	4.222106	2.93E-05	0.000749
CXCL9	0.467113	7.445295	2.774342	0.005761	0.049091
HMGCS2	0.464863	6.837775	2.986254	0.002978	0.030561
TRAC	0.459949	7.148391	6.209976	1.20E-09	1.46E-07

FAM101B	-0.54106	7.047396	-6.87394	2.08E-11	4.24E-09
CRYAB	-0.54226	6.761887	-4.72547	3.07E-06	0.000119
GXYLT2	-0.54277	5.670511	-4.62972	4.79E-06	0.00017
SNAI2	-0.54497	6.909273	-4.6508	4.35E-06	0.000157
DDR2	-0.5457	6.557653	-5.44438	8.55E-08	5.56E-06
ROR2	-0.54686	4.200732	-6.97179	1.12E-11	2.49E-09
PI15	-0.54718	4.539634	-7.22008	2.22E-12	6.32E-10
HS3ST3A1	-0.55001	4.789204	-6.89549	1.82E-11	3.79E-09
PDGFRL	-0.55006	5.302196	-4.99995	8.22E-07	4.01E-05
COL5A3	-0.5503	6.066116	-9.08878	3.17E-18	3.62E-15
MNDA	-0.55207	5.932943	-4.49344	8.92E-06	0.000283
PCDH17	-0.55263	5.886143	-7.33187	1.06E-12	3.23E-10
TIMP1	-0.55293	11.64122	-7.14035	3.75E-12	9.54E-10
TGFB1I1	-0.55455	8.30257	-5.0675	5.89E-07	2.95E-05
GJA1	-0.55597	8.857674	-5.57574	4.25E-08	3.09E-06
MAP1B	-0.55712	5.70212	-6.26221	8.85E-10	1.11E-07
COL8A1	-0.55871	4.706207	-6.52017	1.88E-10	2.87E-08
IBSP	-0.56058	4.149437	-8.52236	2.36E-16	1.83E-13
RAMP1	-0.56175	6.82493	-4.90506	1.31E-06	5.87E-05
COL7A1	-0.56343	6.099693	-8.10404	5.03E-15	3.11E-12
SRPX	-0.56451	6.365058	-3.99408	7.58E-05	0.001645
COL18A1	-0.56612	6.986816	-6.7774	3.84E-11	7.22E-09
TLR2	-0.56716	6.715803	-7.27969	1.50E-12	4.44E-10
CSGALNACT1	-0.57001	6.201585	-5.74893	1.66E-08	1.40E-06
VEGFC	-0.57057	5.60409	-7.07688	5.66E-12	1.35E-09
FSCN1	-0.57574	5.989362	-5.36759	1.28E-07	7.93E-06
ALOX5AP	-0.57584	8.292202	-5.35947	1.33E-07	8.22E-06
HTRA1	-0.57635	8.418398	-5.3897	1.14E-07	7.11E-06
GPR116	-0.57656	6.388797	-7.72795	7.15E-14	3.29E-11
LGALS1	-0.58143	11.20141	-6.71864	5.54E-11	9.91E-09
OSMR	-0.58275	5.079199	-7.50222	3.36E-13	1.19E-10
FERMT2	-0.58359	6.448413	-5.45409	8.12E-08	5.35E-06
COL4A1	-0.58558	9.801283	-6.78237	3.72E-11	7.06E-09
LOXL1	-0.58604	7.141214	-4.92425	1.19E-06	5.42E-05
MME	-0.5888	5.276075	-4.96313	9.85E-07	4.65E-05
C3orf80	-0.58953	3.619016	-6.4238	3.38E-10	4.72E-08
COLEC12	-0.59047	5.904816	-4.52545	7.72E-06	0.000253
GPX8	-0.592	6.260627	-5.92053	6.36E-09	6.29E-07

PAQR8	0.459434	8.023608	5.641476	2.98E-08	2.28E-06
CD27	0.457387	6.258567	5.561881	4.58E-08	3.26E-06
CD79A	0.455801	6.162101	6.357069	5.04E-10	6.61E-08
NKX2-3	0.45516	5.234377	3.950432	9.05E-05	0.001892
SCARA5	0.45391	4.83461	6.937293	1.39E-11	3.05E-09
DHRS11	0.45305	7.044123	3.955369	8.87E-05	0.00186
PSMB9	0.4511	9.175771	5.220168	2.73E-07	1.54E-05
DDX60	0.450553	7.338974	3.947892	9.14E-05	0.001906
ISX	0.449315	6.463148	2.633062	0.008752	0.065694
ATOH1	0.448149	6.034421	4.051438	5.99E-05	0.001346
AQP8	0.447898	4.917102	2.942497	0.003423	0.033697
LINC01207	0.447292	6.471488	2.799833	0.005332	0.046611
FRMD3	0.447052	5.256457	4.594469	5.64E-06	0.000195
NAT1	0.443209	7.601378	4.794548	2.22E-06	8.99E-05
SLC27A2	0.441792	7.371283	4.657159	4.22E-06	0.000153
LOC101929272	0.439266	3.766253	6.174242	1.48E-09	1.75E-07
FAM3D	0.435642	9.326154	2.936709	0.003487	0.034165
C15orf48	0.434761	10.7604	3.432908	0.000652	0.009322
BCAS1	0.429522	6.17197	4.26746	2.41E-05	0.000637
ST6GALNAC1	0.429181	8.866075	2.260301	0.024278	0.128624
PBLD	0.429066	6.299837	3.792608	0.000169	0.003102
GZMK	0.428147	5.454539	3.898421	0.000112	0.002222
FFAR4	0.425826	4.604396	3.341142	0.000904	0.012154
TNFRSF11A	0.425756	6.616343	3.565321	0.000402	0.006306
LCN2	0.421986	10.84694	2.393761	0.017084	0.102451
AIM2	0.419323	5.067519	3.473235	0.000564	0.008283
BTNL8	0.419171	5.686326	3.258304	0.001205	0.015427
LYPD8	0.417321	5.435638	3.787306	0.000173	0.003151
FAM46C	0.416172	6.638206	4.496025	8.81E-06	0.00028
PI3	0.41538	8.528658	2.267987	0.023802	0.127014
LINC00261	0.415018	6.685724	2.388027	0.017349	0.103469
PKIB	0.414333	4.965168	3.007588	0.00278	0.028923
MYO1A	0.413284	7.179771	3.756261	0.000195	0.003502
GPR171	0.41224	4.619028	4.246507	2.64E-05	0.000686
AHCYL2	0.407821	7.085594	4.174483	3.58E-05	0.000892
CXCL11	0.406147	5.357005	1.926733	0.05464	0.21331
C2orf88	0.405909	5.199966	3.77701	0.00018	0.003264
IFI27	0.403539	10.93551	4.51352	8.14E-06	0.000265

NPTX2	-0.5924	5.820855	-3.01255	0.002736	0.028638
SYNM	-0.59312	6.590968	-3.11069	0.001985	0.022573
FLNA	-0.59346	8.581541	-5.50046	6.35E-08	4.34E-06
S100A12	-0.59792	3.969695	-6.75144	4.51E-11	8.28E-09
BNIP3	-0.59803	6.368108	-3.82629	0.000148	0.002775
COL4A2	-0.60074	9.270984	-6.85539	2.34E-11	4.57E-09
MFAP2	-0.60487	7.959743	-5.53413	5.31E-08	3.75E-06
TAGLN	-0.60571	9.220655	-4.99872	8.27E-07	4.02E-05
SPHK1	-0.60647	6.289484	-7.1929	2.65E-12	7.28E-10
COL12A1	-0.6082	6.673962	-6.75508	4.41E-11	8.24E-09
ADAM12	-0.61493	5.022114	-7.99929	1.06E-14	5.90E-12
HSD11B1	-0.61647	5.555599	-5.81619	1.14E-08	1.03E-06
FBN1	-0.61827	6.718912	-5.32388	1.60E-07	9.57E-06
KIAA1462	-0.6209	4.030804	-7.43093	5.44E-13	1.77E-10
PCOLCE	-0.62205	8.299987	-5.86171	8.85E-09	8.30E-07
TUBB6	-0.62345	8.123168	-5.97997	4.54E-09	4.60E-07
S100A9	-0.62452	7.877019	-5.57194	4.34E-08	3.13E-06
COL1A1	-0.6318	9.11477	-6.66887	7.55E-11	1.29E-08
SERPINB2	-0.63236	3.779199	-4.5112	8.23E-06	0.000266
WISP1	-0.63334	4.985106	-7.42973	5.49E-13	1.77E-10
STC1	-0.63428	6.054512	-7.18119	2.87E-12	7.66E-10
POSTN	-0.63536	6.357528	-6.74494	4.70E-11	8.55E-09
COL6A3	-0.64233	10.86249	-5.86852	8.52E-09	8.02E-07
SPARC	-0.646	10.19721	-6.25165	9.42E-10	1.17E-07
RP3-428L16.2	-0.654	5.206283	-5.0232	7.33E-07	3.60E-05
COL6A2	-0.65589	7.877511	-6.8729	2.10E-11	4.24E-09
ITGAM	-0.65735	5.997474	-6.6329	9.43E-11	1.56E-08
NXN	-0.66062	8.123148	-6.03325	3.35E-09	3.52E-07
RPS4Y1	-0.66253	8.158601	-2.18924	0.029091	0.144585
PPBP	-0.66283	4.584621	-3.11907	0.00193	0.022082
NCF2	-0.66421	6.086894	-5.87284	8.32E-09	7.90E-07
COL3A1	-0.66635	10.56359	-5.85388	9.25E-09	8.56E-07
ISLR	-0.66975	6.88106	-5.07752	5.60E-07	2.81E-05
MCEMP1	-0.67013	5.355388	-8.8125	2.66E-17	2.50E-14
TIMP3	-0.6707	8.001459	-5.87113	8.40E-09	7.94E-07
EDNRA	-0.68091	6.282576	-6.89603	1.81E-11	3.79E-09
TNC	-0.69152	5.282072	-6.61589	1.05E-10	1.71E-08
PLN	-0.6982	5.175946	-4.28026	2.28E-05	0.000611

LINC01082	0.402874	4.979632	6.164021	1.57E-09	1.85E-07
CLIC4	-0.40224	7.814057	-4.7931	2.23E-06	9.04E-05
ZFPM2	-0.40242	4.215544	-3.57096	0.000394	0.006203
HTR2B	-0.40558	3.683491	-3.29143	0.001075	0.013998
THBS4	-0.40567	5.91393	-3.28781	0.001089	0.014141
CEL	-0.40587	6.745561	-1.59488	0.111439	0.32683
EMP3	-0.40642	7.950589	-4.33767	1.78E-05	0.000505
FCN1	-0.40754	4.688372	-4.97157	9.45E-07	4.50E-05
MMP14	-0.4087	7.194273	-6.37077	4.64E-10	6.24E-08
CCL7	-0.40907	3.485627	-7.18886	2.73E-12	7.38E-10
EML1	-0.41129	4.48026	-4.69049	3.62E-06	0.000136
RGS16	-0.41134	5.25871	-7.55955	2.28E-13	9.30E-11
GLT8D2	-0.41167	6.526531	-3.83558	0.000143	0.002697
DACT1	-0.41259	6.323695	-3.69031	0.000251	0.00433
RBMS1	-0.4141	7.649626	-4.24532	2.65E-05	0.000688
MYADM	-0.41423	8.516688	-5.57013	4.38E-08	3.15E-06
SELE	-0.41438	4.246299	-3.63217	0.000313	0.005171
CFL2	-0.41461	5.439384	-4.0306	6.53E-05	0.001446
CCDC102B	-0.41497	3.453473	-6.19665	1.30E-09	1.57E-07
IGFBP3	-0.41517	9.024925	-4.22042	2.95E-05	0.000754
CHST11	-0.41621	5.459138	-5.72435	1.90E-08	1.57E-06
ENPEP	-0.417	4.086574	-5.26549	2.17E-07	1.26E-05
MEIS2	-0.41727	6.31697	-3.13347	0.00184	0.021194
APOE	-0.41852	7.785975	-3.86276	0.000129	0.00248
DDIT4	-0.41969	9.922266	-4.63059	4.77E-06	0.00017
VGLL3	-0.42025	3.846766	-5.21561	2.80E-07	1.58E-05
FCER1G	-0.42042	7.492474	-3.66565	0.000276	0.004667
TSPYL5	-0.42131	5.112526	-4.49922	8.69E-06	0.000277
KCNE4	-0.42137	4.52278	-5.88997	7.55E-09	7.27E-07
CD14	-0.42171	8.563106	-4.1658	3.72E-05	0.000922
LOC100132891	-0.42207	3.701816	-7.10906	4.59E-12	1.14E-09
PFKFB3	-0.4221	7.456386	-6.56237	1.46E-10	2.27E-08
CDH5	-0.42229	5.993508	-5.5058	6.18E-08	4.25E-06
FGR	-0.4226	5.79392	-6.44709	2.93E-10	4.21E-08
TSHZ3	-0.42284	5.316179	-5.81337	1.16E-08	1.04E-06
CRISPLD1	-0.42352	3.958239	-3.9471	9.17E-05	0.00191
COL16A1	-0.42383	7.365052	-4.76325	2.57E-06	0.000101
DENND5A	-0.42575	7.382079	-6.03694	3.28E-09	3.46E-07
OLFML2B	-0.70558	7.04361	-6.43706	3.12E-10	4.41E-08
RAI14	-0.70915	7.404763	-8.24277	1.85E-15	1.29E-12
VCAN	-0.713	8.522354	-5.47485	7.28E-08	4.88E-06
LINC01279	-0.7143	5.809662	-4.45346	1.07E-05	0.00033
EGFL6	-0.71499	5.876278	-6.08643	2.47E-09	2.74E-07
GPR84	-0.71739	4.452496	-9.41528	2.43E-19	4.13E-16
ENO2	-0.72598	6.172705	-6.93212	1.44E-11	3.12E-09
CCL18	-0.72958	8.134946	-4.46545	1.01E-05	0.000316
FRMD6	-0.73022	6.849697	-6.18552	1.39E-09	1.67E-07
CNN1	-0.73107	7.473903	-4.41671	1.26E-05	0.000378
GREM1	-0.74329	8.562044	-4.08128	5.30E-05	0.001218
AEBP1	-0.74816	8.396113	-5.94243	5.62E-09	5.58E-07
DFNA5	-0.7492	6.09652	-8.13081	4.15E-15	2.64E-12
COL1A2	-0.75105	10.56118	-5.85602	9.14E-09	8.49E-07
S100A8	-0.75147	5.963796	-7.14963	3.53E-12	9.09E-10
C5orf46	-0.75698	4.584295	-7.13799	3.80E-12	9.58E-10
CYP1B1	-0.78983	4.386179	-6.60812	1.10E-10	1.78E-08
C5AR1	-0.79214	6.606217	-7.43698	5.23E-13	1.77E-10
PRRX1	-0.79603	5.450366	-7.44749	4.87E-13	1.67E-10
COL5A1	-0.79667	8.395363	-6.52271	1.85E-10	2.85E-08
ITGA5	-0.79702	7.634107	-8.14393	3.77E-15	2.48E-12
BGN	-0.80358	7.317706	-6.98969	9.95E-12	2.27E-09
SERPINE1	-0.80385	6.15877	-7.16897	3.11E-12	8.20E-10
LOX	-0.80609	5.230664	-7.76115	5.67E-14	2.73E-11
PXDN	-0.80865	7.698569	-8.92323	1.14E-17	1.23E-14
RAB31	-0.80872	8.827722	-7.08864	5.25E-12	1.26E-09
OLR1	-0.82029	3.967009	-6.7312	5.12E-11	9.24E-09
MXRA5	-0.82398	8.956648	-6.43206	3.21E-10	4.52E-08
FN1	-0.82737	8.341462	-7.5559	2.33E-13	9.36E-11
INHBA	-0.84321	6.479783	-8.86952	1.72E-17	1.69E-14
TMEM45A	-0.85618	6.674318	-6.56797	1.41E-10	2.21E-08
FAP	-0.86232	7.14141	-6.44467	2.98E-10	4.24E-08
IL24	-0.86929	5.034856	-7.38674	7.33E-13	2.27E-10
COL5A2	-0.86995	8.992598	-7.31353	1.20E-12	3.59E-10
GFPT2	-0.87011	5.351724	-8.63836	9.92E-17	8.26E-14
TWIST1	-0.8751	5.000959	-7.6385	1.33E-13	5.80E-11
SLC2A3	-0.88241	7.057337	-8.4138	5.28E-16	3.81E-13
SFRP4	-0.88774	5.788231	-4.88793	1.42E-06	6.31E-05

SLN	-0.426	4.657686	-6.21215	1.19E-09	1.46E-07
PLOD2	-0.4262	8.289959	-4.68806	3.66E-06	0.000136
KCNJ8	-0.42676	5.514642	-5.9595	5.10E-09	5.11E-07
RGS2	-0.42687	8.812261	-3.37199	0.00081	0.011142
CXCR2	-0.42717	5.309411	-5.02641	7.22E-07	3.56E-05
MRC1	-0.42866	7.091926	-3.10744	0.002006	0.022758
WNT5A	-0.42965	7.082995	-4.17089	3.64E-05	0.000904
MMP12	-0.43066	10.15566	-3.22106	0.001369	0.016975
LEPRE1	-0.43293	6.996004	-7.09585	5.01E-12	1.22E-09
FZD10	-0.43315	4.491039	-2.17663	0.030026	0.14744
ECM1	-0.43468	7.704369	-4.88472	1.44E-06	6.39E-05
INHBB	-0.43504	6.512212	-4.45423	1.06E-05	0.000329
CTSL	-0.43557	8.812821	-5.68653	2.33E-08	1.86E-06
SOX11	-0.4359	3.720546	-8.22214	2.14E-15	1.45E-12
TNS1	-0.4374	6.365517	-5.52593	5.55E-08	3.88E-06
MRGPRF	-0.43773	6.117013	-3.52396	0.000468	0.007065
CD109	-0.43774	4.796727	-5.79792	1.26E-08	1.11E-06
RCN3	-0.43878	6.837224	-6.28849	7.57E-10	9.59E-08
TNFSF4	-0.4407	5.089685	-3.89176	0.000115	0.002265
MARCO	-0.44156	5.609155	-6.46453	2.64E-10	3.84E-08
PTX3	-0.44353	3.578956	-4.99957	8.24E-07	4.01E-05
ADAMTS2	-0.44405	6.252564	-6.02867	3.44E-09	3.58E-07
CCL4	-0.44509	7.449986	-4.26043	2.48E-05	0.000652
PDGFRB	-0.44581	7.409084	-4.99516	8.42E-07	4.08E-05
PRR16	-0.44644	4.648843	-6.60563	1.12E-10	1.79E-08
LAMB2	-0.44695	6.522734	-6.05979	2.88E-09	3.08E-07
CDH13	-0.44796	4.499247	-7.90048	2.14E-14	1.13E-11
CCL2	-0.44815	8.053583	-3.5473	0.00043	0.006645
LINC01094	-0.45059	3.558555	-5.63969	3.01E-08	2.29E-06
S100A4	-0.4516	10.06647	-4.0774	5.38E-05	0.001231
TMEM47	-0.4524	6.663309	-3.98354	7.91E-05	0.001696
MAB21L2	-0.45504	5.106904	-3.08902	0.002132	0.023852

COMP	-0.89132	5.613543	-5.32824	1.57E-07	9.41E-06
COL15A1	-0.89471	9.030217	-7.43401	5.33E-13	1.77E-10
SULF1	-0.91115	8.966478	-6.57204	1.37E-10	2.18E-08
GAS1	-0.91183	5.099304	-7.50253	3.36E-13	1.19E-10
CTHRC1	-0.9226	9.52073	-6.08927	2.43E-09	2.71E-07
IL6	-0.92918	6.509763	-6.88605	1.93E-11	3.98E-09
ASPN	-0.93593	6.823036	-5.67546	2.48E-08	1.96E-06
G0S2	-0.93856	7.721946	-8.87472	1.65E-17	1.69E-14
BCAT1	-0.95184	5.503836	-10.5585	1.94E-23	6.99E-20
PPAPDC1A	-0.9617	5.65332	-7.00374	9.09E-12	2.09E-09
CXCL6	-0.98058	4.592003	-6.68129	6.99E-11	1.23E-08
PTGS2	-1.0128	5.618164	-6.3614	4.91E-10	6.48E-08
FNDC1	-1.01548	5.923931	-5.83128	1.05E-08	9.54E-07
PROK2	-1.0481	3.475483	-9.16364	1.77E-18	2.26E-15
SPOCK1	-1.07214	6.76675	-6.68747	6.73E-11	1.19E-08
MMP3	-1.08461	8.504816	-5.10877	4.79E-07	2.47E-05
IL1B	-1.08679	7.582593	-8.71964	5.38E-17	4.85E-14
AQP9	-1.09335	6.051277	-10.3654	9.98E-23	3.09E-19
COL11A1	-1.12891	5.951469	-7.55277	2.38E-13	9.39E-11
CLEC5A	-1.13385	4.032976	-12.008	5.09E-29	3.68E-25
BCL2A1	-1.13985	6.217243	-8.48632	3.09E-16	2.30E-13
THBS2	-1.17801	8.516455	-6.86209	2.25E-11	4.45E-09
SFRP2	-1.24794	7.562794	-5.12964	4.32E-07	2.27E-05
TREM1	-1.30932	5.924568	-12.2419	5.91E-30	6.39E-26
TNFAIP6	-1.31372	6.139348	-9.99192	2.26E-21	6.13E-18
CXCL5	-1.34835	4.767967	-9.41292	2.48E-19	4.13E-16
SPP1	-1.36223	6.134267	-10.573	1.71E-23	6.99E-20
MMP1	-1.39462	9.016112	-6.48053	2.40E-10	3.53E-08
CXCL8	-1.49108	8.879283	-9.34408	4.28E-19	5.79E-16
HCAR3	-1.49569	5.771381	-9.57141	6.98E-20	1.51E-16
COL10A1	-1.52065	6.480727	-7.88935	2.31E-14	1.19E-11
MMP9	-1.52404	8.202232	-15.0703	7.23E-42	1.57E-37

Table S5. KEGG analysis results of immune-related DEGs in CRC.

ID	Description	GeneRatio	BgRatio	*p*Value	*p*.Adjust	*q*Value	Geneid	Count
hsa04974	Protein digestion and absorption	20/212	94/7884	3.74E-13	8.42E-11	7.05E-11	COL10A1/COL11A1/COL15A1/COL5A2/COL5A1/COL1A2/COL3A1/COL6A2/COL6A3/COL1A1/COL12A1/COL4A2/MME/COL4A1/COL18A1/COL7A1/COL5A3/SLC16A10/ACE2/MEP1A	20
hsa04061	Viral protein interaction with cytokine and cytokine receptor	16/212	100/7884	7.77E-09	7.12E-07	5.96E-07	CXCL8/CXCL5/CXCL6/IL6/IL24/CCL18/PPBP/CCL2/CCL4/CXCR2/CCL7/CXCL11/CXCL9/CXCL13/CCL28/CXCL14	16
hsa04512	ECM-receptor interaction	15/212	88/7884	9.49E-09	7.12E-07	5.96E-07	SPP1/THBS2/COMP/FN1/ITGA5/COL1A2/TNC/COL6A2/COL6A3/COL1A1/COL4A2/COL4A1/IBSP/LAMB2/THBS4	15
hsa04657	IL-17 signaling pathway	15/212	93/7884	2.08E-08	1.17E-06	9.78E-07	MMP9/CXCL8/MMP1/CXCL5/IL1B/MMP3/PTGS2/CXCL6/IL6/S100A8/S100A9/MMP13/CCL2/CCL7/LCN2	15
hsa05144	Malaria	11/212	49/7884	4.88E-08	2.19E-06	1.84E-06	CXCL8/THBS2/IL1B/IL6/COMP/TLR2/SDC2/CCL2/SELE/THBS4/KLRB1	11
hsa04060	Cytokine-cytokine receptor interaction	26/212	294/7884	7.16E-08	2.69E-06	2.25E-06	CXCL8/CXCL5/IL1B/CXCL6/IL6/IL24/INHBA/CCL18/PPBP/OSMR/OSM/IL1RN/CCL2/CCL4/TNFSF4/INHBB/CXCR2/CCL7/CXCL11/TNFRSF11A/CD27/CXCL9/CXCL13/CCL28/CXCL14/TNFRSF17	26
hsa04933	AGE-RAGE signaling pathway in diabetic complications	14/212	100/7884	3.86E-07	1.24E-05	1.04E-05	CXCL8/IL1B/IL6/FN1/SERPINE1/COL1A2/COL3A1/COL1A1/COL4A2/COL4A1/VEGFC/NOX4/CCL2/SELE	14
hsa05146	Amoebiasis	14/212	102/7884	4.95E-07	1.39E-05	1.17E-05	CXCL8/IL1B/IL6/FN1/COL1A2/COL3A1/ITGAM/COL1A1/COL4A2/COL4A1/TLR2/LAMB2/CD14/MUC2	14
hsa04510	Focal adhesion	19/212	199/7884	1.47E-06	3.66E-05	3.07E-05	SPP1/THBS2/COMP/FN1/ITGA5/COL1A2/TNC/COL6A2/COL6A3/COL1A1/COL4A2/FLNA/COL4A1/VEGFC/IBSP/PDGFC/LAMB2/PDGFRB/THBS4	19
hsa04145	Phagosome	16/212	152/7884	2.87E-06	6.46E-05	5.41E-05	THBS2/COMP/OLR1/ITGA5/NCF2/ITGAM/TUBB6/COLEC12/TLR2/FCGR3B/FCGR2B/MARCO/CTSL/MRC1/CD14/THBS4	16
hsa05134	Legionellosis	9/212	56/7884	1.57E-05	0.000321	0.000269	CXCL8/IL1B/IL6/ITGAM/BNIP3/TLR2/HSPA6/CD14/CASP1	9
hsa00140	Steroid hormone biosynthesis	9/212	60/7884	2.79E-05	0.000523	0.000438	CYP1B1/HSD11B1/DHRS11/UGT2B15/HSD11B2/AKR1C3/UGT2A3/HSD17B2/UGT2B17	9
hsa05133	Pertussis	10/212	76/7884	3.24E-05	0.000561	0.000469	CXCL8/CXCL5/IL1B/CXCL6/IL6/ITGA5/ITGAM/CD14/CFL2/CASP1	10
hsa05323	Rheumatoid arthritis	11/212	93/7884	3.61E-05	0.000581	0.000486	CXCL8/MMP1/CXCL5/IL1B/MMP3/CXCL6/IL6/TLR2/CCL2/CTSL/TNFRSF11A	11
hsa04151	PI3K-Akt signaling pathway	23/212	354/7884	7.31E-05	0.001097	0.000919	SPP1/THBS2/IL6/COMP/FN1/ITGA5/COL1A2/TNC/COL6	23

							A2/COL6A3/COL1A1/COL4A2/COL4A1/OSMR/VEGFC/TLR2/IBSP/OSM/PDGFC/LAMB2/PDGFRB/DDIT4/THBS4	
hsa05205	Proteoglycans in cancer	16/212	204/7884	0.000114	0.001609	0.001348	MMP9/TWIST1/FN1/ITGA5/COL1A2/TIMP3/COL1A1/FLNA/TLR2/PLAU/SDC2/LUM/PLAUR/CTSL/FZD10/WNT5A	16
hsa04062	Chemokine signaling pathway	15/212	189/7884	0.000165	0.002179	0.001824	CXCL8/CXCL5/CXCL6/CCL18/PPBP/CCL2/CCL4/CXCR2/FGR/CCL7/CXCL11/CXCL9/CXCL13/CCL28/CXCL14	15
hsa04668	TNF signaling pathway	11/212	112/7884	0.000199	0.002488	0.002084	MMP9/CXCL5/IL1B/MMP3/PTGS2/CXCL6/IL6/VEGFC/CCL2/SELE/MMP14	11
hsa04640	Hematopoietic cell lineage	10/212	97/7884	0.000261	0.003092	0.00259	IL1B/IL6/ITGA5/ITGAM/MME/CD14/CD2/CD38/CD3D/CD8A	10
hsa05204	Chemical carcinogenesis	9/212	82/7884	0.000331	0.003728	0.003122	PTGS2/CYP1B1/HSD11B1/NAT1/UGT2B15/NAT2/UGT2A3/ADH1C/UGT2B17	9
hsa05165	Human papillomavirus infection	19/212	330/7884	0.001384	0.014828	0.012418	SPP1/THBS2/PTGS2/COMP/FN1/ITGA5/COL1A2/TNC/COL6A2/COL6A3/COL1A1/COL4A2/COL4A1/IBSP/LAMB2/PDGFRB/FZD10/WNT5A/THBS4	19
hsa04620	Toll-like receptor signaling pathway	9/212	104/7884	0.001868	0.019101	0.015996	CXCL8/SPP1/IL1B/IL6/TLR2/CCL4/CD14/CXCL11/CXCL9	9

Table S6. Results of univariate Cox regression analysis.

	GSE39582 Cohort		TCGA Cohort	
	**HR (95% CI)**	***p***	**HR (95% CI)**	***p***
Immunoscore*	1.72(1.25,2.35)	< 0.001	2.40(1.56,3.68)	< 0.001
Age*	1.04(1.02–1.06)	< 0.001	1.03(1.01,1.05)	0.003
Tumor stage		< 0.001		< 0.001
Stage I	1.00(reference)		1.00(reference)	
Stage II	1.54(0.55,4.31)	0.407	1.72(0.65,4.51)	0.274
Stage III	1.86(0.67,5.17)	0.236	2.69(1.02,7.04)	0.045
Stage IV	7.21(2.36,21.97)	< 0.001	6.95(2.69,17.97)	< 0.001
TP53 mutation	1.25(0.84,1.86)	0.268	0.67(0.42,1.09)	0.815

*Continuous variable. Abbreviation: HR, hazard ratio; CI, confidence interval.

Table S7. Results of multivariable Cox regression analysis.

	GSE39582 Cohort		TCGA Cohort	
	**HR (95% CI)**	***p***	**HR (95% CI)**	***p***
Immunoscore*	1.76(1.26,2.46)	< 0.001	1.95(1.20,3.18)	0.007
Age*	1.04(1.02–1.06)	< 0.001	1.04(1.02,1.06)	< 0.001
Tumor stage		< 0.001		< 0.001
I	1.00(reference)		1.00(reference)	
II	1.35(0.48,3.79)	0.567	1.72(0.65,4.53)	0.273
III	1.59(0.57,4.45)	0.374	2.83(1.06,7.53)	0.037
IV	6.34(2.07,19.43)	0.001	6.96(2.64,18.36)	< 0.001
TP53 mutation		0.876		0.096
$TP53^{W}$	1.00(reference)		1.00(reference)	
$TP53^{MT}$	1.09(0.72,1.67)	0.684	0.67(0.42,1.09)	0.103

*Continuous variable. Abbreviation: HR, hazard ratio; CI, confidence interval.

Table S8. The difference of proportion of immune cell types in low and high score CRC groups.

Immune Cell Types	*p*-Value
B cells naive	0.0117
B cells memory	0.0564
Plasma cells	0.0023
T cells CD8	0.0265
T cells CD4 naive	0.0960
T cells CD4 memory resting	< 0.0001
T cells CD4 memory activated	0.6003
T cells follicular helper	0.5257
T cells regulatory (Tregs)	0.9363
T cells gamma delta	0.9524
NK cells resting	0.25
NK cells activated	0.6367
Monocytes	0.0011
Macrophages M0	< 0.0001
Macrophages M1	0.0083
Macrophages M2	0.0114
Dendritic cells resting	0.7255
Dendritic cells activated	0.1437
Mast cells resting	0.3106
Mast cells activated	0.0443
Eosinophils	0.02762
Neutrophils	0.3409
